# *Shox2* is required for the proper development of the facial motor nucleus and the establishment of the facial nerves

**DOI:** 10.1186/s12868-015-0176-0

**Published:** 2015-07-09

**Authors:** Jessica M Rosin, Deborah M Kurrasch, John Cobb

**Affiliations:** 1 Department of Biological Sciences, University of Calgary, 2500 University Drive N.W., BI286D, Calgary, AB T2N 1N4 Canada; 2 Department of Medical Genetics, Alberta Children’s Hospital Research Institute, University of Calgary, 3330 Hospital Drive N.W., Room HS2275, Calgary, AB T2N 4N1 Canada

**Keywords:** Short stature homeobox 2 (*Shox2*) gene, Islet 1 (*Isl1*), Sonic hedgehog (*Shh*), Visceral motor neurons (vMNs), Facial motor nucleus, Facial (VII) nerves

## Abstract

**Background:**

Axons from the visceral motor neurons (vMNs) project from nuclei in the hindbrain to innervate autonomic ganglia and branchial arch-derived muscles. Although much is known about the events that govern specification of somatic motor neurons, the genetic pathways responsible for the development of vMNs are less well characterized. We know that vMNs, like all motor neurons, depend on sonic hedgehog signaling for their generation. Similarly, the paired-like homeobox 2b (*Phox2b*) gene, which is expressed in both proliferating progenitors and post-mitotic motor neurons, is essential for the development of vMNs. Given that our previous study identified a novel role for the short stature homeobox 2 (*Shox2*) gene in the hindbrain, and since SHOX2 has been shown to regulate transcription of islet 1 (*Isl1)*, an important regulator of vMN development, we sought to determine whether *Shox2* is required for the proper development of the facial motor nucleus.

**Results:**

Using a *Nestin*-*Cre* driver, we show that elimination of *Shox2* throughout the brain results in elevated cell death in the facial motor nucleus at embryonic day 12.5 (E12.5) and E14.5, which correlates with impaired axonal projection properties of vMNs. We also observed changes in the spatial expression of the vMN cell fate factors *Isl1* and *Phox2b*, and concomitant defects in *Shh* and *Ptch1* expression in *Shox2* mutants. Furthermore, we demonstrate that elimination of *Shox2* results in the loss of dorsomedial and ventromedial subnuclei by postnatal day 0 (P0), which may explain the changes in physical activity and impaired feeding/nursing behavior in *Shox2* mutants.

**Conclusions:**

Combined, our data show that *Shox2* is required for development of the facial motor nucleus and its associated facial (VII) nerves, and serves as a new molecular tool to probe the genetic programs of this understudied hindbrain region.

**Electronic supplementary material:**

The online version of this article (doi:10.1186/s12868-015-0176-0) contains supplementary material, which is available to authorized users.

## Background

In the hindbrain, motor neuron progenitors are born in a region-specific manner, neighboring the floor plate, in response to their level of exposure to different morphogenetic gradients [1, 2]. These neuronal progenitors differentiate in a rhombomere-specific pattern, which requires the correct spatiotemporal expression of the homeotic (*Hox*) gene clusters [3–6]. Motor neuron progenitors in the hindbrain can differentiate into somatomotor neurons (sMNs), which innervate the skeletal muscles of the body, or visceromotor neurons (vMNs), which innervate autonomic ganglia (general vMNs) and branchial arch-derived muscles (special vMNs) [3, 7, 8]. Facial (VII) motor neurons, which originate from rhombomeres 4/5, belong to the vMN class of motor neurons. At embryonic day 11.25 (E11.25), facial motor neurons begin to migrate caudally to the pial side of the hindbrain to form the facial motor nucleus, which subsequently segregates into two lobes [6, 9, 10]. Despite the elucidation of the genetic mechanisms that specify sMN identity, the corresponding pathways responsible for the establishment of vMNs are less well understood. Furthermore, even though sonic hedgehog (SHH) signaling is known to be required for the development of all motor neurons [7, 11–15], the upstream regulators of *Shh* expression and function during late embryonic and early postnatal development, especially within the vMNs of the facial motor nucleus, remain largely unknown [16].

The paired-like homeobox 2b (*Phox2b*) gene is expressed in both proliferating progenitors and post-mitotic motor neurons and is required for the development of vMNs [17, 18]. Similarly, the homeodomain transcription factor islet1 (ISL1), which is expressed in both post-mitotic sMNs and vMNs, plays a broad role in motor neuron identity and specification [7, 8, 17, 19, 20]. In the absence of *Isl1,* mice die during embryogenesis around E11.5. This embryonic lethality is associated with an increase in cell death in the hindbrain and neural tube, suggesting that loss of *Isl1* function results in the death of cells that were fated to differentiate into motor neurons [19]. In the trigeminal and dorsal root ganglia (DRG), *Isl1* appears to play distinct roles during early (i.e. neurogenesis) versus late (i.e. subtype specification) development [21]. In cranial ganglia, *Isl1* is required for cell survival and *Isl1* mutants show thinner blunted facial (VII) ganglia [22]. However, the precise role of *Isl1* in these cell types and in their respective hindbrain nuclei, such as the facial motor nucleus, remains poorly documented.

The adult facial motor nucleus contains seven subnuclei; the lateral, dorsolateral, dorsal intermediate, ventral intermediate, dorsomedial and ventromedial nuclei are located within the main nucleus, while the seventh, the dorsal accessory nuclei, is located above the main nucleus [23, 24]. Neurons innervating the nasolabial musculature are located in the lateral facial motor nucleus, while neurons supplying the auricular musculature are located in the medial facial motor nucleus [23, 24]. Together the lateral, intermediate and medial subnuclei comprise 43, 27.1, and 28.7%, respectively, of the motor neurons in the adult facial motor nucleus, with the dorsal accessory nuclei contributing the final 1.2% [23].

Studies with mouse knockout models have shown that *Shox2* is required for normal development of the humerus and femur [25–27], the anterior palate [28], the temporomandibular joint of the jaws [29], the sinoatrial valves and pacemaker region of the heart [30, 31], and TrkB-positive mechanosensory neurons of the dorsal root ganglia [32]. Most recently, *Shox2* has been shown to play an important role in development of the inferior colliculus and cerebellum [33]. We suspected a function for *Shox2* during facial motor nucleus development given our previous study demonstrating a novel role for *Shox2* in the hindbrain [33], and since SHOX2 has been shown to regulate transcription of *Isl1* [34], an important regulator of vMN development [7, 8, 19, 22]. In the current study we show that conditional inactivation of *Shox2* in the brain results in impaired axonal projections of vMNs and a concomitant loss of medially located neurons in the facial motor nucleus postnatally. Furthermore, we demonstrate that *Shox2*-mutant neonates display impaired feeding behavior, perhaps due to facial paralysis as a result of improper development and function of the facial nerves.

## Results

### *Shox2* expression in the face and facial motor nucleus during development

**Figure 1:**
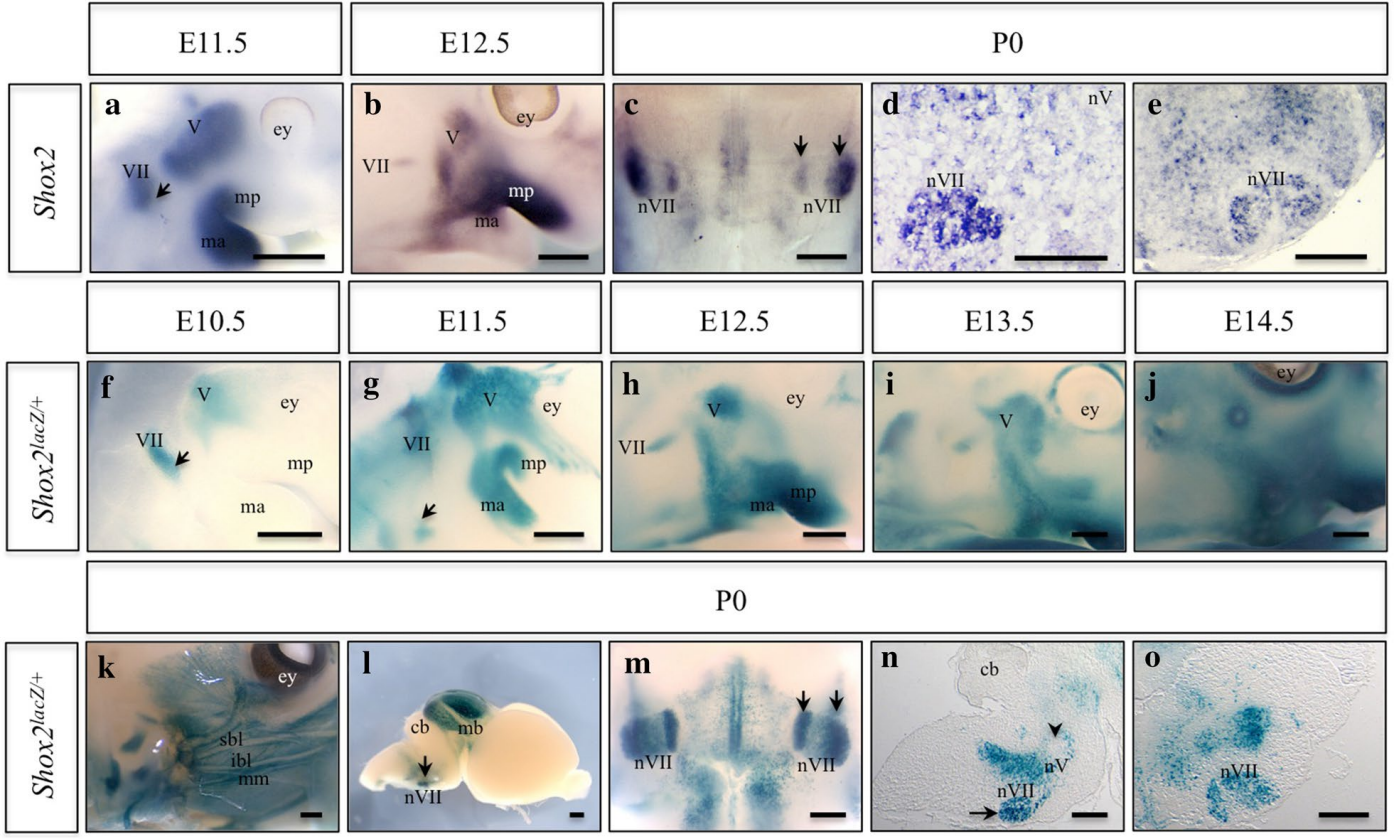
*Shox2* mRNA and *lacZ* expression in the developing embryonic face and postnatal facial motor nucleus. **a**, **b** Whole-mount in situ hybridization (WISH) at E11.5 (**a**) and E12.5 (**b**) show *Shox2* expression in the trigeminal (*V*) and facial (*VII*, *arrow*) ganglia, in addition to the developing maxillary process (mp) and mandibular arch (ma). **c**–**e** In situ hybridization (ISH) on whole P0 brains (**c**, ventral viewpoint) and sagittal (**d**) or coronal (**e**) sections through the brain show *Shox2* expression in the facial motor nucleus (*nVII*, *arrows*). **f**–**j** X-gal staining of *Shox2* ^*lacZ/*+^ embryos at E10.5 (**f**), E11.5 (**g**), E12.5 (**h**), E13.5 (**i**), and E14.5 (**j**) shows that *Shox2* is expressed in the trigeminal and facial ganglion (**f**, **g**, *arrow*), in addition to the developing maxillary process and mandibular arch. Starting at E12.5 and continuing to E14.5, *Shox2* expression becomes restricted to the trigeminal ganglion and the facial mesenchyme that will contribute to the maxilla and mandible, however it is absent from the facial ganglia. **k**–**o** X-gal staining of *Shox2* ^*lacZ/*+^ pups in the P0 face (**k**), brain (**l**, side viewpoint; **m**, ventral viewpoint) and sagittal (**n**) or coronal (**o**) sections through the brain show *Shox2* expression in the tissue surrounding the facial nerves (**k**), with the exception of the nerves themselves, and *Shox2* expression in the facial motor nucleus (**l**–**o**, *arrows*). *ey* eye, *nV* trigeminal nucleus, *sbl* superior buccolabial nerve, *ibl* inferior buccolabial nerve, *mm* marginal mandibular nerve, *cb* cerebellum, *mb* midbrain. *Scale bar* 500 μm.

Whole-mount in situ hybridization (WISH) at E11.5 showed *Shox2* expression in the trigeminal (V) and facial (VII) ganglia, in addition to the developing maxillary process and mandibular arch (Figure 1a). *Shox2* continued to be expressed in the trigeminal (V) ganglia, maxillary process and mandibular arch at E12.5 (Figure 1b). We also visualized the *Shox2* expression pattern with a novel *Shox2* ^*lacz*^ allele (described in “Methods”). X-gal staining of embryos carrying the *Shox2* ^*lacz*^ allele accurately reproduced the endogenous *Shox2* expression pattern with increased sensitivity and less background compared to WISH (Additional file 1: Figure S1). At E10.5, *Shox2* ^*lacZ/*+^ embryos showed *lacZ* staining in the trigeminal (V) and facial (VII) ganglia of the embryo (Figure 1f). Similarly at E11.5*, lacZ* staining was visible in the trigeminal (V) and facial (VII) nerves of the embryo, in addition to the maxillary process and mandibular arch (Figure 1g). *LacZ* continued to be expressed in the trigeminal (V) ganglion of the embryonic face from E12.5 to E13.5, and could be seen in the developing pharyngeal arch (Figure 1h, i). At E14.5 *lacZ* expression was present in the facial mesenchyme that contributes to the maxilla and mandible (Figure 1j). However, from E12.5 onward *lacZ* staining was no longer observed in the facial (VII) ganglia (Figure 1h–j). Postnatally (P0) *lacZ* expression was maintained in the facial mesenchyme surrounding the facial (VII) nerves, but not within the axons themselves (Figure 1k). Examination of *Shox2* expression early embryonically in the developing brain showed *Shox2* staining in a region of post-mitotic neurons adjacent to the floor plate (Additional file 2: Figure S2A–B), a region where *Phox2b*+*/Isl1*+ are localized (Additional file 2: Figure S2C–E) [18]. Later in the P0 brain, *Shox2* was expressed in the facial motor nucleus (Figure 1c–e). Similarly, in *Shox2* ^*lacZ/*+^ animals, *lacZ* staining was strongly expressed in both the lateral and medial lobes of the facial motor nucleus (Figure 1l–o); however, staining was not visible within the trigeminal nucleus (Figure 1n, arrowhead).

### *Nestin-CreShox2*-mutant pups exhibit defects in feeding behavior

To determine the functional role of *Shox2* in the facial motor nucleus, we used *Nestin*-*Cre* [35] to delete the *Shox2* gene conditionally in neural tissue throughout the central nervous system (CNS). We crossed *Nestin*-*Cre*; *Shox2* ^+*/*−^ mice with *Shox2* ^*flox/flox*^ mice [25] to obtain conditional mutant *Nestin*-*Cre*; *Shox2* ^*flox/*−^ embryos and neonates. *Shox2* expression was eliminated in the brain of *Nestin*-*Cre* *Shox2*-mutant embryos, including the facial motor nucleus (Additional file 3: Figure S3C–D, dashed-circle). All *Nestin*-*Cre Shox2*-mutant pups died between 18 to 26 h following birth. This limited our characterization of *Shox2* function to embryonic and neonatal stages. P0 *Nestin*-*Cre*; *Shox2* ^*flox/*−^ mutant pups displayed reduced activity and impaired feeding behavior as early as 6 h following birth (Additional file 4: Movie 1), which correlated with a lack of milk in the stomachs of *Nestin*-*Cre*; *Shox2* ^*flox/*−^ mutant pups (Additional file 3: Figure S3E, arrows). Since *Shox2* is required for the proper development of the palate [28], we analyzed the anterior and posterior palate of *Nestin*-*Cre*; *Shox2* ^*flox/*−^ mutant pups. We did not observe any obvious defects or malformations in the palate of *Nestin*-*Cre*; *Shox2* ^*flox/*−^ mutant pups (Additional file 3: Figure S3F–K), suggesting that the absence of milk in *Shox2*-mutant neonatal stomachs was not due to cleft palate malformations. *Nestin*-*Cre*; *Shox2* ^*flox/*−^ mutant pups also displayed tremors 16–26 h following birth (Additional file 4: Movie 1), which could be related to defects in neural circuitry or result from a loss of *Shox2* function in other regions of the hindbrain.

### Facial (VII) nerve branching defects in *Nestin-Cre Shox2*-mutant animals

**Figure 2:**
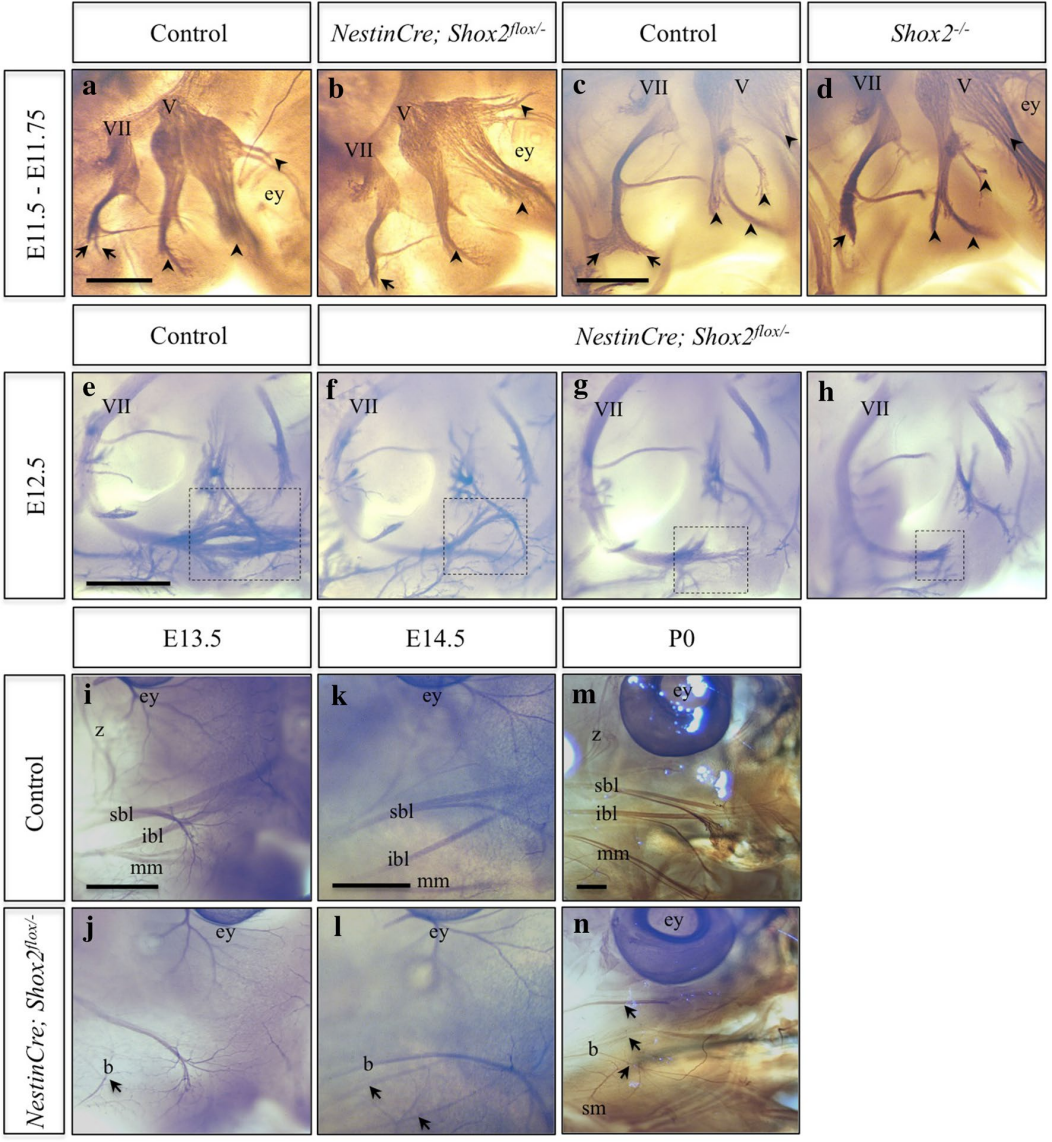
Facial nerve truncations in *Shox2*-mutant animals. **a**–**d** Side view of the E11.5 (**a**, **b**) or E11.75 (**c**, **d**) face of control (**a**, **c**), *Nestin*-*Cre; Shox2* ^*flox*/−^ (**b**) and *Shox2* ^−/−^ (**d**) embryos stained with the 2H3 anti-neurofilament antibody. At E11.5 the main branch of the facial nerve (*VII*) begins to separate into smaller projections in control embryos (**a**, *arrows*), while a single branch is visible in *Nestin*-*Cre; Shox2* ^*flox*/−^ conditional knockout embryos (compare **a** to **b**, *arrows*). At E11.75 the facial nerve is noticeably truncated in the *Shox2* ^−/−^ embryos (compare **c** to **d**, *arrows*), while the trigeminal nerve (*V*; **a**–**d**, *arrowheads*) remains intact. **e**–**h** Side view of the E12.5 face of control (**e**) and *Nestin*-*Cre; Shox2* ^*flox*/−^ (**f**–**h**) embryos stained with the 2H3 antibody display the range in severity of facial nerve truncation, with **f** being the least severe truncation and** h** being the most severe truncation observed in *Nestin*-*Cre; Shox2* ^*flox*/−^ embryos (compare **e** to **f**–**h**, *dashed-box*). (**i**–**n**) Side view of the E13.5 (**i**, **j**), E14.5 (**k**, **l**) and P0 (**m**, **n**) face of control (**i**, **k**, **m**) and *Nestin*-*Cre; Shox2* ^*flox*/−^ (**j**, **l**, **n**) animals stained with the 2H3 antibody. At E13.5 and E14.5 the zygomatic (z), superior buccolabial (sbl), inferior buccolabial (ibl) and marginal mandibular (mm) nerve branches can be seen in control (**i**, **k**) embryos but are not visible in *Nestin*-*Cre; Shox2* ^*flox*/−^ (**j**, **l**) embryos (compare **i** to **j** and **k** to **l**, *arrows*). At P0 the truncated remnants of what appears to be the superior buccolabial nerve were observable in *Nestin*-*Cre; Shox2* ^*flox*/−^ pups (compare **m** to **n**, *arrows*). *ey* eye, *b* buccal nerve, *sm* superficial masseter nerve. *Scale bar* 500 μm.

Since *Shox2*-mutant neonates displayed impaired feeding but an apparently normal palate, we hypothesized that *Shox2* mutants fail to nurse properly due to facial paralysis. To test this hypothesis, we first sought to determine if the abnormal feeding behavior resulted from the loss of a subset of axons in the nerves of the face. We stained control and *Nestin*-*Cre Shox2*-mutant embryos and P0 heads with the 2H3 antibody that visualizes nerves by detecting neurofilaments (Figure 2). In E11.5 *Nestin*-*Cre; Shox2* ^*flox/*−^ conditional knockout embryos the trigeminal nerves (V) appeared normal (Figure 2a–b, arrowheads). To confirm that the trigeminal nerves (V) were intact even in the complete absence of *Shox2*, we performed neurofilament staining on E11.75 *Shox2*-null embryos (Figure 2c–d, arrowheads) [25]. While the trigeminal nerves (V) remained intact, the facial nerves (VII) were truncated and failed to branch properly in both *Nestin*-*Cre; Shox2* ^*flox/*−^ conditional knockout and *Shox2*-null embryos (Figure 2a–d, arrows). At E12.5 the facial (VII) nerves continued to be truncated as compared to controls (Figure 2e–h, dashed-box). Neurofilament staining at E12.5 displayed a range in severity of facial (VII) nerve truncation, with Figure 2f showing the least severe truncation and Figure 2h having the most severe truncation we observed among seven embryos. Later at E13.5 (Figure 2i), E14.5 (Figure 2k), and early postnatal (P0; Figure 2m) stages, the zygomatic, superior buccolabial, inferior buccolabial and marginal mandibular nerve branches of the facial (VII) nerve could be seen in controls (Figure 2i, k, m) [36], however these distinct facial (VII) nerve branches were truncated or absent in *Nestin*-*Cre; Shox2* ^*flox/*−^ mutant animals (Figure 2j, l, n; arrows). Similar to E12.5, facial (VII) nerve truncation ranged in severity at the later embryonic and early postnatal time-points analyzed (data not shown). The severity in facial (VII) nerve truncation observed in *Nestin*-*Cre; Shox2* ^*flox/*−^ mutant animals, displayed in Figure 2j, l, n, are representative of the most common truncations observed.

**Figure 3:**
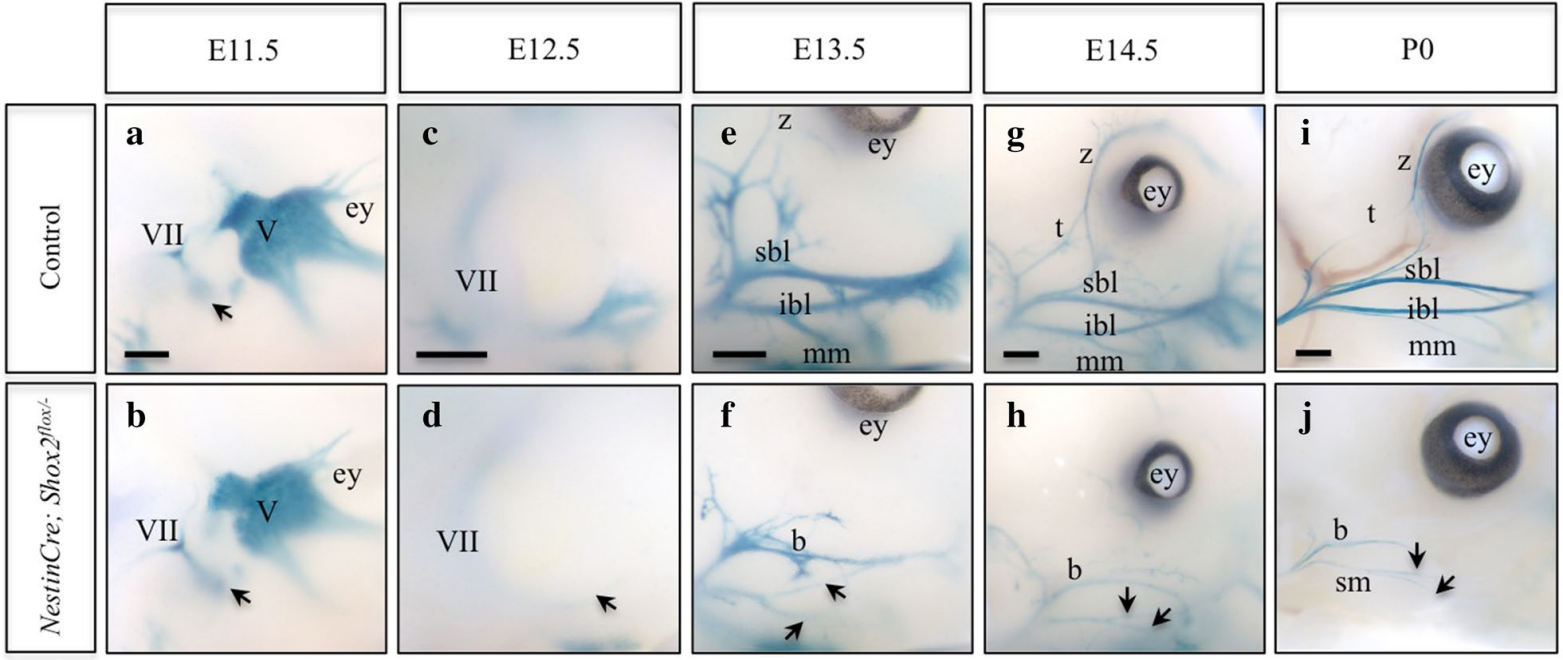
*LacZ* staining highlights truncated facial nerves and impaired axonal projection properties of vMNs in mice lacking *Shox2* function. **a**–**j** BAC RP23-105B3-*lacZ* transgenic animals stained with X-gal. **a**, **b** Side view of the E11.5 face of control (**a**) and *Nestin*-*Cre; Shox2* ^*flox*/−^ (**b**) embryos display similar axonal projections of the main branch of the facial nerve (*VII*) (compare **a** to **b**, *arrows*). **c**, **d** Side view of the E12.5 face of control (**c**) and *Nestin*-*Cre; Shox2* ^*flox*/−^ (**d**) embryos display facial nerve truncation in mice lacking *Shox2* function (compare **c** to **d**, *arrow*). **e**–**h** Side view of the E13.5 (**e**, **f**) and E14.5 (**g**, **h**) face of control (**e**, **g**) and *Nestin*-*Cre; Shox2* ^*flox*/−^ (**f**, **h**) embryos highlights the zygomatic (z), temporal (t), superior buccolabial (sbl), inferior buccolabial (ibl) and marginal mandibular (mm) nerve branches in control (**e**, **g**) embryos, which are truncated or lost in *Nestin*-*Cre; Shox2* ^*flox*/−^ embryos (compare **e** to **f** and **g** to **h**, *arrows*). **i**, **j** Side view of the P0 face of control (**i**) and *Nestin*-*Cre; Shox2* ^*flox*/−^ (**j**) pups demonstrate that the facial nerves are absent in *Nestin*-*Cre; Shox2* ^*flox*/−^ pups (compare **i** to **j**, *arrows*). *ey* eye, *b* buccal nerve, *sm* superficial masseter nerve. *Scale bar* 500 μm.

To confirm the persistent lack of proper facial (VII) nerve branching and development, particularly at late embryonic and early postnatal time-points when the larger head size led to confounding background staining, we used a second transgenic line, this one carrying a bacterial artificial chromosome (BAC) containing the *Shox2* gene with a *lacZ* insertion (previously described BAC RP23-105B3-*lacZ* transgenic line [37]) to visualize the facial (VII) nerves. While obvious facial (VII) nerve truncations were difficult to visualize at E11.5 (Figure 3a, b), the main branch of the facial (VII) nerve was noticeably truncated, as the smaller ganglionic projections were absent at E12.5 in *Nestin*-*Cre; Shox2* ^*flox/*−^ mutant embryos as compared to controls (Figure 3c–d, arrow). Furthermore, consistent with our neurofilament staining, at later embryonic (E13.5 and E14.5) and early postnatal (P0) stages, the zygomatic, temporal, superior buccolabial, inferior buccolabial and marginal mandibular nerve branches of the facial (VII) nerve were observed in controls (Figure 3e, g, i) and appeared truncated or absent in *Nestin*-*Cre; Shox2* ^*flox/*−^ mutant animals (Figure 3f, h, j; arrows). For example, by P0 the facial (VII) nerves appeared to be absent in *Nestin*-*Cre; Shox2* ^*flox/*−^ mutant animals (Figure 3j, arrows). The buccal and superficial masseter nerves, both sensory components originating from the trigeminal (V) nerve [36], were the only nerves visible in *Nestin*-*Cre; Shox2* ^*flox/*−^ mutant animals, leaving the face almost completely devoid of innervation (Figure 3j, arrows). To determine whether the facial (VII) nerve disruptions occurred as a result of the slight reduction in *Shox2* expression in the embryonic facial mesenchyme (Additional file 3: Figure S3A–B, arrows), or whether this was related specifically to disruptions in the facial motor nucleus, we conditionally removed *Shox2* in all neural crest derivatives using a *Wnt1*-*Cre* driver. This resulted in the complete loss of *Shox2* expression in the face (Additional file 5: Figure S4A, B), and ultimately did not appear to result in any disruptions to the development of the facial (VII) nerves (Additional file 5: Figure S4C, D). Together these results show impaired axonal projection properties of visceral motor neurons (vMNs) in the facial motor nucleus of the hindbrain in mice lacking *Shox2*.

### Loss of *Shox2* in the facial motor nucleus results in elevated apoptosis

**Figure 4:**
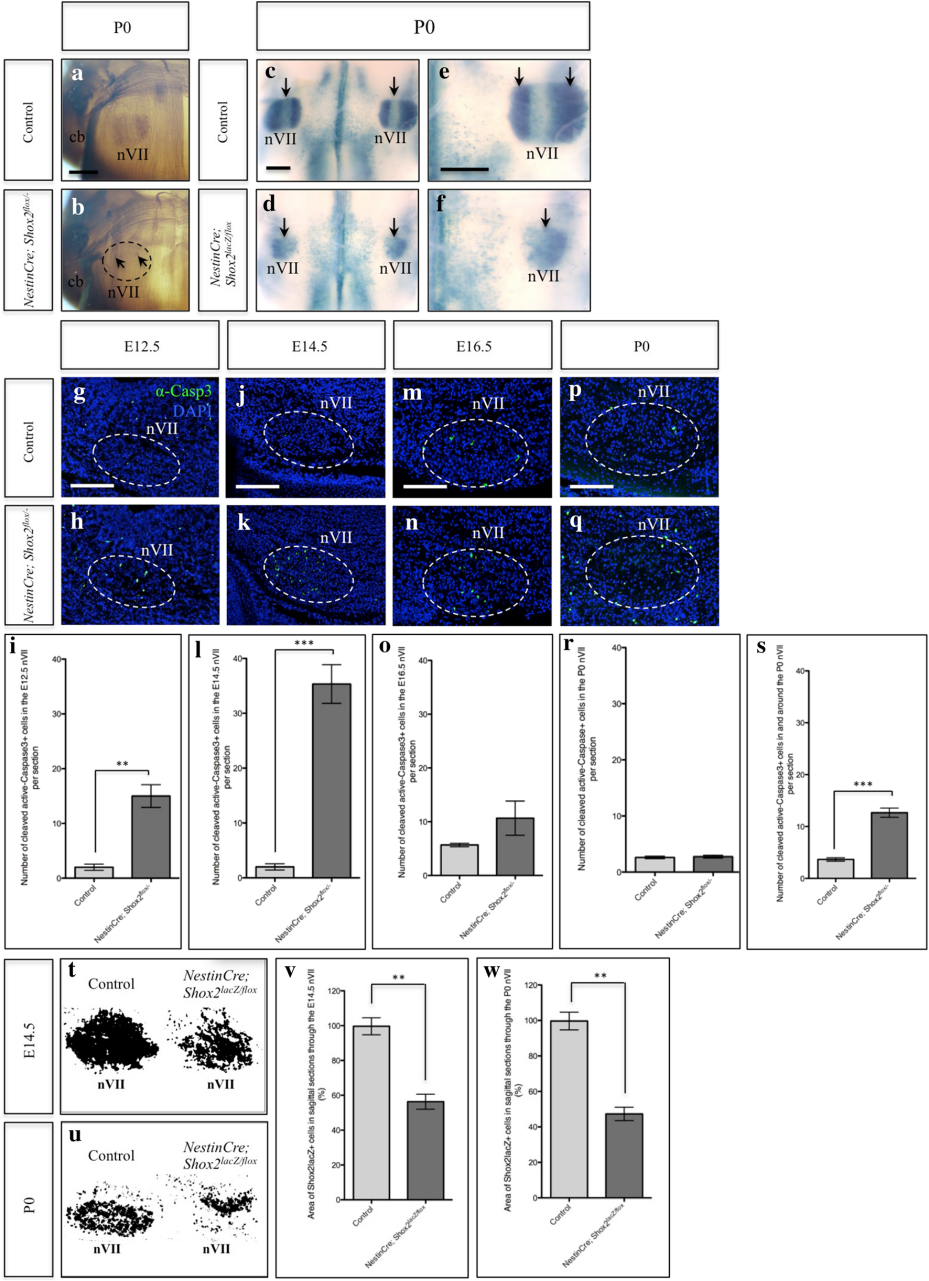
*Nestin*-*Cre* driven conditional *Shox2*-deletion results in disruptions in the facial motor nucleus and changes in cell death. **a**, **b** Ventral view of the P0 brain of control (**a**) and *Nestin*-*Cre; Shox2* ^*flox*/−^ (**b**) animals stained with the 2H3 antibody demonstrate that the facial motor nucleus (*nVII*) is severely reduced in *Nestin*-*Cre; Shox2* ^*flox*/−^ pups (compare **a** to **b**, *dashed-circle* and *arrows*). **c**–**f** X-gal staining of P0 control (**c**, **e**) and *Nestin*-*Cre; Shox2* ^*lacZ/flox*^ (**d**, **f**) pup brains (viewed ventrally) show *Shox2* expression in the facial motor nucleus and demonstrate that the nucleus is severely reduced in size (compare **e** to **f**, *arrows*) in mice lacking *Shox2* function. Cleaved active-CASP3 immunostaining on E12.5 (**g**, **h**), E14.5 (**j**, **k**), E16.5 (**m**, **n**) and P0 (**p**, **q**) control (**g**, **j**, **m**, **p**) and *Nestin*-*Cre; Shox2* ^*flox/*−^ mutant (**h**, **k**, **n**, **q**) sagittal sections through the facial motor nucleus (*nVII*). **i** Quantification of the number of cleaved active-CASP3 + cells present in the E12.5 facial motor nucleus (see **g**, **h**) of control and *Nestin*-*Cre; Shox2* ^*flox/*−^ embryos (plotted values are the mean ± S.E.M, 3 sections per animals, n = 3 animals, p = 0.0038). **l** Quantification of the number of cleaved active-CASP3 + cells present in the E14.5 facial motor nucleus (see **j**, **k**) of control and *Nestin*-*Cre; Shox2* ^*flox/*−^ embryos (plotted values are the mean ± S.E.M, 3 sections per animals, n = 3 animals, p = 0.0007). **o** Quantification of the number of cleaved active-CASP3 + cells present in the E16.5 facial motor nucleus (see **m**, **n**) of control and *Nestin*-*Cre; Shox2* ^*flox/*−^ embryos (plotted values are the mean ± S.E.M, 3 sections per animals, n = 3 animals, p = 0.1929). **r** Quantification of the number of cleaved active-CASP3 + cells present in the P0 facial motor nucleus (see **p**, **q**, *dashed-circle*) of control and *Nestin*-*Cre; Shox2* ^*flox/*−^ embryos (plotted values are the mean ± S.E.M, 3 sections per animals, n = 3 animals, p = 0.6875). **s** Quantification of the number of cleaved active-CASP3 + cells present in and around the P0 facial motor nucleus (see **p**, **q**, entire panel including cells within the *dashed-circle*) of control and *Nestin*-*Cre; Shox2* ^*flox/*−^ embryos (plotted values are the mean ± S.E.M, 3 sections per animals, n = 3 animals, p = 0.0007). **t**, **u** Representative binary particle area diagrams from ImageJ displaying *Shox2lacZ* + cell staining at E14.5 (**t**) and P0 (**u**) in controls and *NestinCre; Shox2* ^*lacZ/flox*^ facial motor nuclei. **v** Quantification of the area of *Shox2lacZ* + cells present in sagittal sections through the center of the E14.5 facial motor nucleus (see **t**) of control and *Nestin*-*Cre; Shox2* ^*lacZ/flox*^ embryos (plotted values are the mean ± S.E.M, control = 3, *Nestin*-*Cre; Shox2* ^*lacZ/flox*^ = 3, p = 0.0027). **w** Quantification of the area of *Shox2lacZ* + cells present in sagittal sections through the center of the P0 facial motor nucleus (see **u**) of control and *Nestin*-*Cre; Shox2* ^*lacZ/flox*^ animals (plotted values are the mean ± S.E.M, control = 3, *Nestin*-*Cre; Shox2* ^*lacZ/flox*^ = 3, p = 0.0011). *cb* cerebellum. **a**–**f** *Scale bar* 500 μm. **g**–**q** *Scale bar* 250 μm.

Since *Shox2*-mutant embryos and neonates display impaired axonal projection properties and were unable to nurse properly, we next wanted to determine if development of the facial motor nucleus was disrupted in the absence of *Shox2*. Both neurofilament and *lacZ* staining of P0 control and *Nestin*-*Cre; Shox2* ^*flox*/−^ or *Nestin*-*Cre; Shox2* ^*lacZ/flox*^ pup brains demonstrated that the facial motor nucleus was severely reduced in size, with disruptions in neuronal spatial organization in mice lacking *Shox2* function (Figure 4a–f, arrows; note that the *Shox2* ^*lacZ*^ allele is a null or severely hypomorphic allele, see “Methods”). While the symmetry between the left and right facial motor nuclei appears to be relatively maintained (Figure 4c–d, arrows), the two lobes that normally comprise the facial motor nucleus are no longer present (Figure 4e–f, arrows). We next investigated whether an increase in apoptosis could account for the changes observed in the facial motor nucleus of *Shox2*-mutant brains. We found an increase in the number of apoptotic cells present at E12.5 and E14.5 in the facial motor nucleus of *Shox2*-mutants (Figure 4g–l); however, no significant increase in apoptosis was detected in the E16.5 or P0 facial motor nucleus of *Shox2*-mutant animals (Figure 4m–r). We observed a ~7.5-fold increase in the number of cleaved active-CASP3 + cells in the E12.5 facial motor nucleus of *Nestin*-*Cre; Shox2* ^*flox/*−^ animals as compared to controls (Figure 4i; control n = 3, *Nestin*-*Cre; Shox2* ^*flox/*−^ n = 3, p = 0.0038). We also observed a striking ~17.7-fold increase in the number of cleaved active-CASP3 + cells in the E14.5 facial motor nucleus of *Nestin*-*Cre; Shox2* ^*flox/*−^ animals as compared to controls (Figure 4l; control n = 3, *Nestin*-*Cre; Shox2* ^*flox/*−^ n = 3, p = 0.0007). While the slight elevation in apoptosis detected in the E16.5 and P0 facial motor nucleus of *Shox2*-mutant animals was not significantly different from controls (Figure 4o, r), we did observe a ~3.5-fold increase in the number of cleaved active-CASP3 + cells at P0 when we included the tissue immediately surrounding the facial motor nucleus of *Nestin*-*Cre; Shox2* ^*flox/*−^ animals (Figure 4p–q, entire panel including cells within the dashed-circle) in our cell count (Figure 4s; control n = 3, *Nestin*-*Cre; Shox2* ^*flox/*−^ n = 3, p = 0.0007). Moreover, when we calculated the area of *Shox2lacZ* + cells remaining in sagittal sections through the center of the facial motor nucleus of *Nestin*-*Cre; Shox2* ^*lacZ/flox*^ animals as compared to controls at E14.5 (Figure 4t) and P0 (Figure 4u), we observed a ~44% decrease in the area of *Shox2lacZ* + cells in the E14.5 facial motor nucleus (Figure 4v; control n = 3, *Nestin*-*Cre; Shox2* ^*lacZ/flox*^ n = 3, p = 0.0027) and a ~53% decrease in the area of *Shox2lacZ* + cells in the P0 facial motor nucleus (Figure 4w; control n = 3, *Nestin*-*Cre; Shox2* ^*lacZ/flox*^ n = 3, p = 0.0011). Therefore, the higher levels of apoptosis are correlated with a decrease in the size of the facial motor nucleus, and taken together, the results demonstrate that apoptosis contributes to the disruptions observed in the facial motor nucleus of *Nestin*-*Cre; Shox2* ^*flox/*−^ animals.

### *Shox2* is required for normal expression of markers of vMN identity in the facial motor nucleus

**Figure 5:**
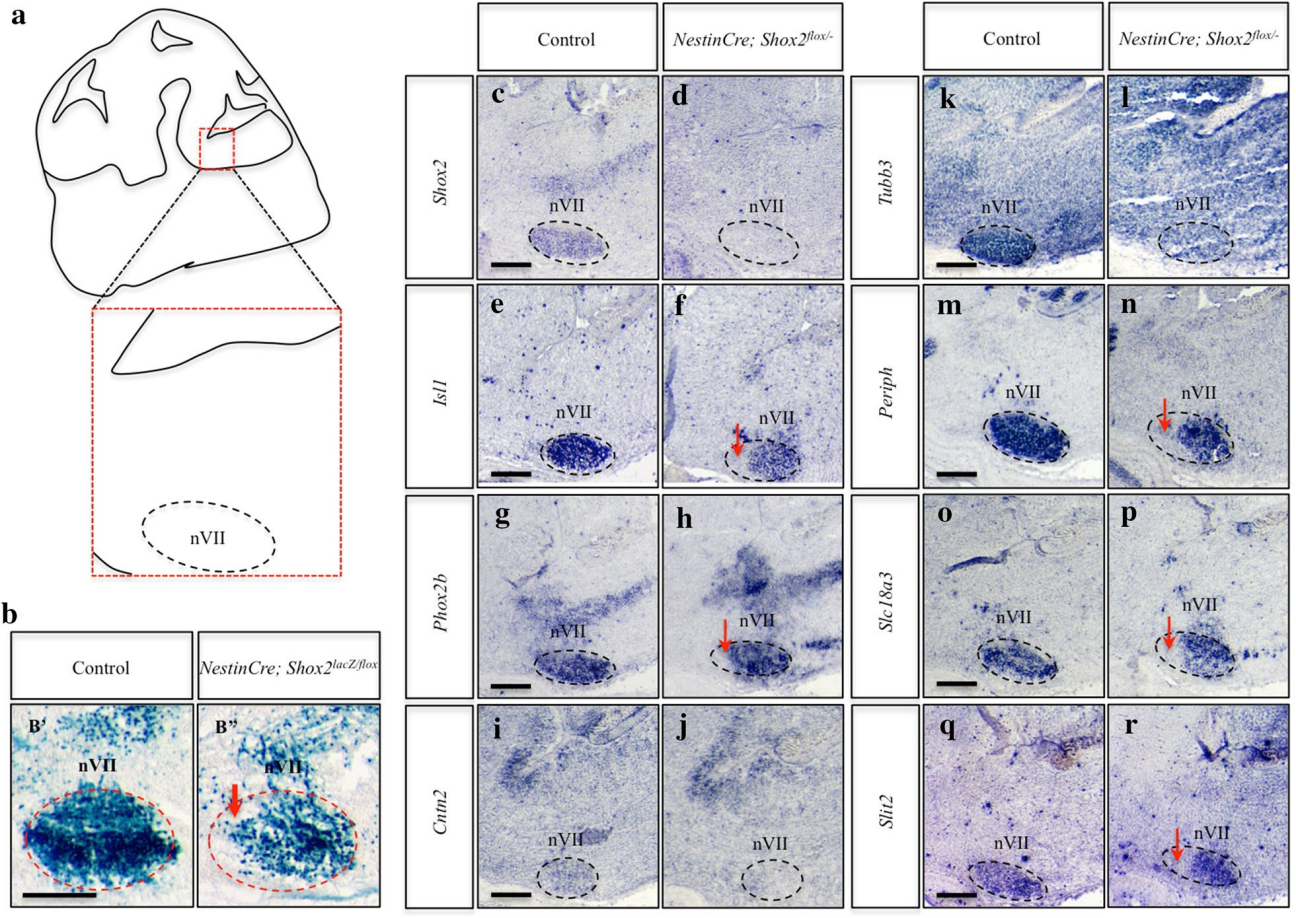
Loss of *Shox2* in the brain results in subtle changes in the expression pattern of *Isl1* and *Phox2b* in the E14.5 facial motor nucleus. **a** Diagram of a sagittal section through the E14.5 brain highlights the regions represented in **b**–**r**. **b** X-gal stained sagittal sections through E14.5 embryos shows decreased staining in the facial motor nucleus (*nVII*) of *Nestin*-*Cre; Shox2* ^*lacZ/flox*^ (*B*″) embryos that lack *Shox2* function, as compared to controls (*B*′). **c**–**r** ISH on E14.5 control (**c**, **e**, **g**, **i**, **k**, **m**, **o**, **q**) and *Nestin*-*Cre; Shox2* ^*flox/*−^ (**d**, **f**, **h**, **j**, **l**, **n**, **p**, **r**) sagittal sections shows loss of *Shox2* expression (compare **c** to **d**, *dashed-circle*), and subtle changes in the spatial expression patterns of *Isl1* (compare **e** to **f**, *arrow*), *Phox2b* (compare **g** to **h**, *arrow*), *Cntn2* (compare **i** to **j**, *dashed-circle*), *Tubb3* (compare **k** to **l**, *dashed-circle*), *Periph* (compare **m** to **n**, *arrow*), *Slc18a3* (compare **o** to **p**, *arrow*) and *Slit2* (compare **q** to **r**, *arrow*) in the developing facial motor nucleus. The *dashed-circles* in paired panels being compared (e.g. *B*′ and *B*″) are the same size, and this applies from *B*′ to **r**. *Scale bar* 250 μm.

**Figure 6:**
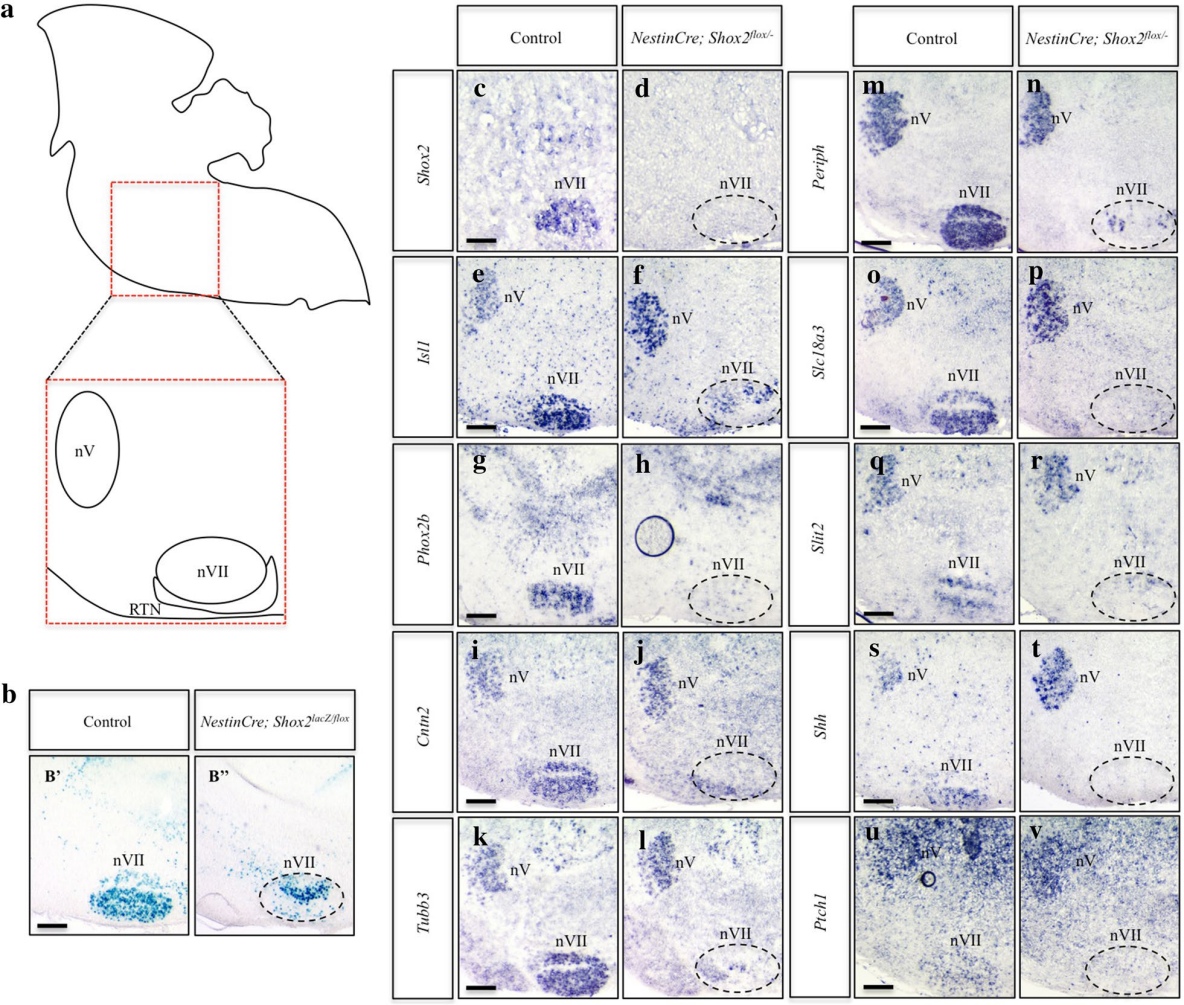
*Shox2* is required for the proper development of the facial motor nucleus. **a** Diagram of a sagittal section through the P0 brain highlights the regions represented in **b**–**v**. **b** X-gal stained sagittal sections through P0 brains show a decrease in staining levels in the facial motor nucleus (*nVII*) of *Nestin*-*Cre; Shox2* ^*lacZ/flox*^ (*B*″) animals as compared to controls (*B*′). **c**–**v** ISH on P0 control (**c**, **e**, **g**, **i**, **k**, **m**, **o**, **q**, **s**, **u**) and *Nestin*-*Cre; Shox2* ^*flox/*−^ (**d**, **f**, **h**, **j**, **l**, **n**, **p**, **r**, **t**, **v**) sagittal sections shows loss of *Shox2* expression (compare **c** to **d**, *dashed-circle*), and decreases in *Isl1* (compare **e** to **f**, *dashed-circle*), *Phox2b* (compare **g** to **h**, *dashed-circle*), *Cntn2* (compare **i** to **j**, *dashed-circle*), *Tubb3* (compare **k** to **l**, *dashed-circle*), *Periph* (compare **m** to **n**, *dashed-circle*), *Slc18a3* (compare **o** to **p**, *dashed-circle*), *Slit2* (compare **q** to **r**, *dashed-circle*), *Shh* (compare **s** to **t**, *dashed-circle*) and *Ptch1* (compare **u** to **v**, *dashed-circle*) expression in the facial motor nucleus. *nV* trigeminal nucleus, *RTN* retrotrapezoid nucleus. *Scale bar* 250 μm.

Given the marked reduction in size of the facial motor nucleus in *Shox2* mutants (Figure 4), we next sought to investigate whether factors other than facial (VII) nerve truncation and cell death could be changing in the facial motor nucleus following the loss of *Shox2.* Prior to E14.5, we did not observe any obvious changes in gene expression or disruptions to the migratory patterns of vMNs (Additional file 2: Figure S2F-K, M-R). At E14.5, however, sagittal sections of the facial motor nucleus showed reductions in the overall size of the nucleus in *Nestin*-*Cre; Shox2* ^*lacZ/flox*^ embryos (Figure 5b), which was consistent with the regional increase in the number of cleaved active-CASP3 + cells at this time-point (Figure 4k). Since SHOX2 has been previously shown to regulate *Isl1* [34], an important regulator of vMN development [7, 19, 22], we next examined the expression of *Isl1* in *Shox2*-mutant animals. We observed a regionally restricted loss of *Isl1* and the hindbrain vMN determinant *Phox2b* in the facial motor nucleus as early as E14.5 in *Nestin*-*Cre; Shox2* ^*flox/*−^ mutant embryos as compared to controls (Figure 5e–h, arrows). Since loss of *Isl1* function in the trigeminal and DRG have been shown to result in changes in the expression of contactin 2 (*Cntn2*), tubulin β-3 chain (*Tubb3*) and peripherin 1 (*Periph*) [38], and given that *Phox2b*-deficient mice have been used to identify the facial motor nucleus as a source of *Slit homolog 2* (*Slit2*) signaling [39], we next examined the expression patterns of these genes. Although subtle changes in the spatial patterns and expression levels of *Cntn2* and *Tubb3* were observed, *Periph1*, solute carrier family 18 (*Slc18a3*) and *Slit2* phenocopied the regionally specific absence of *Isl1* and *Phox2b* in the facial motor nucleus at E14.5 in *Nestin*-*Cre; Shox2* ^*flox/*−^ mutant embryos (Figure 6i–r, dashed-circle and arrows). The changes in expression for these genes continued to be observed between *Nestin*-*Cre; Shox2* ^*flox/*−^ mutant embryos and controls at E16.5 (data not shown). Together, these results show that *Shox2* is required for normal expression of markers of vMN identity at E14.5.

### Loss of *Shox2* results in severe developmental disruptions to the facial motor nucleus

In contrast to the subtle changes at E14.5, postnatally we observed a drastic reduction in the size of the facial motor nucleus and changes to the overall organization of motor neurons in *Nestin*-*Cre; Shox2* ^*flox/*−^ mutant animals (Figure 6), consistent with the phenotype displayed in Figure 4. Specifically, *lacZ* staining of P0 control and *Nestin*-*Cre; Shox2* ^*lacZ/flox*^ sagittal sections of the facial motor nucleus demonstrate that the nucleus is reduced in size in mice lacking *Shox2* (Figure 6b). The expression levels of *Isl1*, *Phox2b*, *Cntn2*, *Tubb3*, *Periph*, *Slc18a3* and *Slit2* were all strikingly reduced in the P0 facial motor nucleus of *Nestin*-*Cre; Shox2* ^*flox/*−^ mutant animals as compared to controls (Figure 6e–r, dashed-circle). Since conditional inactivation of *Shox2* in the brain was previously shown to result in the down-regulation of *Shh* expression in dorsal-residing Purkinje cells of the cerebellum [33], we next investigated whether loss of *Shox2* function in the facial motor nucleus interfered with the expression of *Shh*. The expression of both *Shh* and patched 1 (*Ptch1*) were absent in the facial motor nucleus of *Nestin*-*Cre; Shox2* ^*flox/*−^ mutant animals (Figure 6s–v).

**Figure 7:**
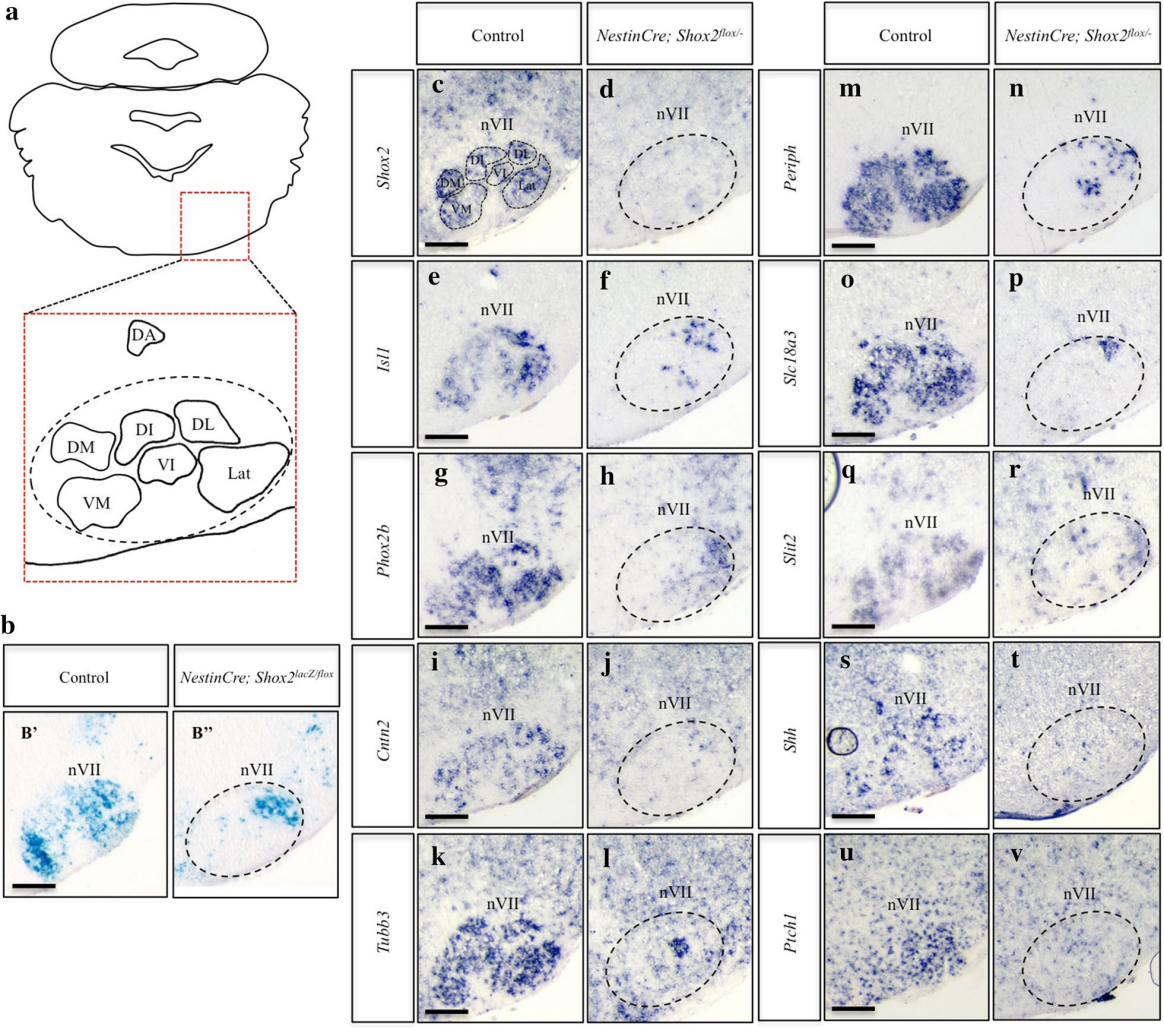
Loss of *Shox2* function in the facial motor nucleus interferes with the proper development of the medial subnuclei. **a** Diagram of a coronal section through the P0 brain highlights the region represented in panels **b**–**v**, particularly the 7 neuronal clusters that form the facial motor nucleus (*nVII*, *dashed-circle*) (image adapted from [23]). **b** X-gal stained coronal sections through P0 brains shows a loss of medial neuronal clusters in the facial motor nucleus of *Nestin*-*Cre; Shox2* ^*lacZ/flox*^ (*B*″) brains as compared to controls (*B*′). **c**–**v** ISH on P0 control (**c**, **e**, **g**, **i**, **k**, **m**, **o**, **q**, **s**, **u**) and *Nestin*-*Cre; Shox2* ^*flox/*−^ (**d**, **f**, **h**, **j**, **l**, **n**, **p**, **r**, **t**, **v**) coronal sections shows loss of *Shox2* expression (compare **c** to **d**, *dashed-circle*), and decreases in *Isl1* (compare **e** to **f**, *dashed-circle*), *Phox2b* (compare **g** to **h**, *dashed-circle*), *Cntn2* (compare **i** to **j**, *dashed-circle*), *Tubb3* (compare **k** to **l**, *dashed-circle*), *Periph* (compare **m** to **n**, *dashed-circle*), *Slc18a3* (compare **o** to **p**, *dashed-circle*), *Slit2* (compare **q** to **r**, *dashed-circle*), *Shh* (compare **s** to **t**, *dashed-circle*) and *Ptch1* (compare **u** to **v**, *dashed-circle*) expression in the facial motor nucleus. *DA* dorsal accessory, *DM* dorsomedial, *VM* ventromedial, *DI* dorsal intermediate, *VI* ventral intermediate, *DL* dorsolateral, *Lat* lateral. *Scale bar* 250 μm.

Although the loss of *Shox2* in the brain resulted in drastic reductions in the size of the facial motor nucleus and the expression of the above-mentioned genes, the spared vMNs remaining in the facial motor nucleus of *Shox2* mutants appeared to be mostly localized to the dorsal regions of the nucleus (Figure 6B″, f, n). This suggested that there could be a region-specific loss of distinct subnuclei in the facial motor nucleus in *Shox2*-mutant animals. Therefore we examined the expression patterns of these same genes in coronal sections of the facial motor nucleus, as this would allow us to more clearly observe the six subnuclei that comprise the main facial motor nucleus; specifically the lateral, dorsolateral, dorsal intermediate, ventral intermediate, dorsomedial and ventromedial nuclei [23, 24]. *LacZ* staining of P0 control and *Nestin*-*Cre; Shox2* ^*lacZ/flox*^ coronal sections of the facial motor nucleus demonstrated that elimination of *Shox2* resulted in the loss of medially-localized subnuclei, specifically the dorsomedial and ventromedial nuclei, in addition to severe disruptions to the development of the intermediate nuclei, especially the dorsal intermediate (Figure 7b). Similar to what we observed at P0 in sagittal sections of the facial motor nucleus (Figure 6), the expression of *Isl1*, *Phox2b*, *Cntn2*, *Tubb3*, *Periph*, *Slc18a3*, *Slit2, Shh* and *Ptch1* were restricted to the lateral or intermediate regions in coronal sections of the facial motor nucleus, or completely lost in *Nestin*-*Cre; Shox2* ^*flox/*−^ mutant animals as compared to controls (Figure 7e–v, dashed-circle). While the regional changes in expression were variable between the genes examined, in general the dorsomedial, ventromedial and dorsal intermediate subnuclei of the facial motor nucleus appeared to be most strongly affected in *Shox2*-mutant animals (Figure 7), which may reflect a regional specific requirement for *Shox2* in the facial motor nucleus or may indicate that there are populations of vMNs within the facial motor nucleus that do not express *Shox2* during development. Together, the results suggest that *Shox2* is required for the development of specific subcompartments within the broader facial motor nucleus.

## Discussion

In this study, we used a *Nestin*-*Cre* driver and a conditional knockout strategy to determine the contribution of *Shox2* to the development of the facial motor nucleus. We found that mice lacking *Shox2* function failed to develop proper vMNs in the medial subnuclei normally present in the facial motor nucleus, which was accompanied by truncated or absent facial (VII) nerve projections. Our results have uncovered an unexpected and novel role for *Shox2* in the development of the facial motor nucleus and facial (VII) nerves. Specifically, *Shox2* is required for cell survival and perhaps plays a region-specific role in the development and/or maintenance of normal transcriptional programs of vMNs of the facial motor nucleus. Ultimately, in the absence of *Shox2,* pups lack facial (VII) nerves and display abnormal feeding behavior.

### *Shox2* is required for cell survival in the facial motor nucleus

*Nestin*-*Cre*-mediated deletion of *Shox2* in the brain resulted in a significant increase in the number of apoptotic cells present at E12.5 and E14.5 in the facial motor nucleus of *Shox2*-mutants. Given that truncated and abnormal facial (VII) nerve projections could be observed in *Nestin*-*Cre; Shox2* ^*flox/*−^ mutants from E11.5 onward, apoptosis could: (1) contribute to the disruptions in the axonal projection properties of vMNs in the facial motor nucleus, or (2) result from a lack of peripheral trophic support for the facial (VII) nerves in *Shox2* mutants. Similarly, the changes observed in the size of the P0 facial motor nucleus also likely results from elevated cell death embryonically in *Shox2*-mutant animals. However, given that there are ~6,400 neurons that comprise the facial motor nucleus [23, 24], future analysis is needed to quantify cell death accurately over a developmental time course to determine if the levels of cell death we observe in *Shox2*-mutant animals is sufficient to account for the disruptions observed in the facial motor nucleus in the absence of *Shox2*. This is similar to what has been documented in *Isl1* mutants, as loss of *Isl1* is associated with increased cell death in the trigeminal, DRG, hindbrain and neural tube prior to E14.5, while later developmentally *Isl1* functions during subtype specification [19, 21, 22, 38]. Accordingly, if *Shox2* is required to maintain proper *Isl1* expression in vMNs of the facial motor nucleus, similar to what has been observed during heart development [34], and *Isl1* is required to induce the subsequent differentiation of adjacent neurons, similar to what has been observed in the neural tube [19], this could explain the elevated cell death observed in neurons adjacent to the facial motor nucleus at P0. Pattyn et al. [7] have shown that unlike PHOX2B and ISL1, NKX6 proteins (NKX6.1 and NKX6.2) are not required for the establishment of vMN identity, yet NKX6 proteins are necessary to prevent vMNs from trans-differentiating into improper neuronal subtypes. Similar to *Shox2* mutants, mice lacking *Nkx6* function display impaired axonal projection properties of vMNs and facial nerve disruptions [7]. Together, the *Shox2*-mutant model could provide an additional example to support the argument of Pattyn et al. that vMN differentiation requires multiple inputs rather than one dominant determinant [7]. Alternatively, necrosis in the facial motor nucleus region could be responsible for the observed ectopic increase in the number of apoptotic cells in tissue immediately surrounding the facial motor nucleus.

### *Shox2* may play a regional specific role in the transcriptional programs of vMNs during development of the facial motor nucleus

In this study we have shown that *Shox2* is expressed in the facial motor nucleus and provide evidence that *Shox2* plays an important role during facial motor nucleus development. We have demonstrated that loss of *Shox2* function results in the down-regulation or loss of *Isl1*, *Phox2b*, *Cntn2*, *Tubb3*, *Periph*, *Slc18a3* and *Slit2* in the facial motor nucleus. Since loss of *Isl1* function in the trigeminal and DRG have been shown to result in changes in the expression of *Cntn2*, *Tubb3* and *Periph* [38], and given that *Phox2b*-deficiency influences *Slit2* expression [39], we propose that the observed changes in gene expression in *Shox2* mutants could result from direct or indirect regulation of *Isl1* and/or *Phox2b* by SHOX2. Since *Isl1* can rescue *Shox2*-mediated bradycardia, and *Isl1* has been shown to be a transcriptional target of SHOX2 in vitro [34], it would not be surprising if SHOX2 regulates *Isl1* in the facial motor nucleus.

As conditional inactivation of *Shox2* in the brain was previously shown to result in the down-regulation of *Shh* expression in dorsal-residing Purkinje cells of the cerebellum [33], we also investigated whether loss of *Shox2* function in the facial motor nucleus interfered with expression of *Shh*. Similar to what was observed in the cerebellum, the expression of both *Shh* and *Ptch1* were reduced or lost in the facial motor nucleus of *Shox2*-mutant animals, suggesting that *Shox2* acts upstream of SHH signaling, or that the neurons that would normally express *Shh* underwent apoptosis earlier embryonically in *Shox2*-mutant animals. Despite the known critical function of *Shh* in motor neuron generation [7, 11–15], the role of *Shh* in the later development of the vMNs of the facial motor nucleus remains largely unknown. In rats, SHH appears to play a role in the regeneration of the facial motor nucleus and facial (VII) nerves following nerve injury [16]. Therefore, as the facial motor nucleus normally undergoes massive cell death from E19 to P10 [24], the late-onset of *Shh* expression in the facial motor nucleus could be localized to specific vMNs for cell survival. This hypothesis will be tested in future studies. Together, it appears that *Shox2* is necessary for proper *Isl1* and *Phox2b* expression levels in the developing facial motor nucleus, and is subsequently required for late-onset *Shh* expression; whether this results from facial (VII) nerve disruption and embryonic apoptosis in *Shox2* mutants or occurs via direct or indirect regulation of *Isl1*, *Phox2b* and/or *Shh* has yet to be determined.

Since the phenotype observed in the facial motor nucleus of *Shox2* mutants, including the changes in gene expression, were most prominent in the medial and intermediate subnuclei, factors other than SHOX2 must contribute to the development and survival of the lateral subnuclei of the facial motor nucleus. The observed phenotype could reflect a regional specific requirement for *Shox2* in the facial motor nucleus or indicate that there are populations of vMNs within the facial motor nucleus that do not express *Shox2*. The latter hypothesis is supported by results depicted in Figure S2F, H, J, as the *Shox2* expression domain appears more restricted then those of *Isl1* and *Phox2b*; therefore, future analysis will be required to examine whether SHOX2−/ISL1+ and/or SHOX2−/PHOX2B+ cell populations exist in the facial motor nucleus. Alternatively, if vMNs destined for the lateral subnuclei of the facial motor nucleus are specified first, this could explain why they are the least disrupted group of nuclei apparent in *Shox2* mutants. However, considering the severe disruptions to facial (VII) nerve development observed in *Shox2* mutants, including those that innervate the nasolabial musculature that are supplied by neurons located in the lateral subnuclei of the facial motor nucleus [23, 24], it is also possible that *Shox2* is required for the proper development of the lateral facial motor nuclei. This is further supported by changes in the expression of *Cntn2*, *Tubb3*, *Shh* and *Ptch1* in the lateral facial motor nucleus. Taken together, it appears that *Shox2* may play a role in the transcriptional programs of vMNs during development of the facial motor nucleus; whether this occurs via a regional requirement for *Shox2* in the facial motor nucleus or influences the development of the entire facial motor nucleus in an equal manner has yet to be determined.

### Disruptions in the development of the facial motor nucleus influences the axonal projections of vMNs of the facial (VII) nerves in *Shox2*-mutant animals

As *Shox2*-mutant neonates display impaired feeding behavior, we wondered if this was due to a loss of a subset of axons in the nerves present in the face or whether it reflected a more substantial innervation deficit. Our findings demonstrate that the loss of *Shox2* causes severe truncations and disruptions to the development of the facial (VII) nerves, which could result from: (1) a lack of peripheral trophic support for the facial (VII) nerves, (2) vMN cell death, (3) facial motor nucleus defects that influence the axonal projections of vMNs and target acquisition of the facial (VII) nerves, or a combination thereof. It should be mentioned, however, that disruptions in peripheral trophic support for the facial (VII) nerves would have to result from *Shox2’s* autonomous function in the facial (VII) nerves themselves, and not as a result of *Shox2* playing a role as a peripheral target, since loss of *Shox2* expression in the face in *Wnt1*-*Cre; Shox2* ^*flox/*−^ mutant animals does not disrupt facial (VII) nerve development.

Given that the face of *Shox2*-mutant neonates are severely devoid of innervation, the behavioral abnormalities exhibited by *Shox2*-mutant animals could be attributed to facial paralysis. Disruption of *Hoxb1* function has been shown to result in facial nerve defects in mice, where the phenotype is similar to that of human patients with Bell’s Palsy or Moebius Syndrome [40]. Similarly, other *Hox*-mutant mouse models have displayed disruptions in facial (VII) nerve development, which correlate with facial impairments [36]. Moreover, *Isl1* mutants display disruptions in the development of the facial (VII) nerves, however similar to *Shox2* mutants, early postnatal lethality has limited the behavioral analysis of these animals [21, 22, 38]. To determine if disruption to the facial motor nucleus is the cause of the early postnatal lethality of *Nestin*-*Cre; Shox2* ^*flox/*−^ animals, future studies could employ more restricted hindbrain *Cre* lines to specifically remove *Shox2* expression from the facial motor nucleus. If regionally restricted loss of *Shox2* in facial motor nucleus does not result in postnatal lethality, future studies could examine *Shox2*-mutant behavior later postnatally and in the adult to test whether *Shox2*-mutant animals have control over their face or are experiencing facial paralysis.

## Conclusions

Taken together, these results demonstrate that elimination of *Shox2* in the brain results in disruptions in the development of the facial (VII) nerves and the facial motor nucleus. The present study demonstrates that in the absence of *Shox2* the murine face develops with severe innervation deficits, ultimately highlighting the importance of *Shox2* to proper facial motor nucleus development. The *Shox2*-mutant model can now be exploited to further our understanding of normal neuronal development and circuit formation; processes that are likely dysregulated in human facial paralysis disorders.

## Methods

### Mice

To generate conditional *Shox2* knockout animals, mice bearing the *Nestin*-*Cre* transgene [35], in addition to a wild-type and deleted *Shox2* allele, were crossed to mice carrying two floxed *Shox2* alleles [25]. *Shox2* mutants were positive for *Nestin*-*Cre* and carried one deleted and one floxed *Shox2* allele. Littermate control animals carried either one or two functional copies of *Shox2*, as no changes in the gross morphology of the facial motor nucleus or facial nerves were observed in heterozygotes. We designated embryonic day 0.5 (E0.5) to be noon on the day a plug was detected. Mice were maintained on a mixed C57BL/6-129/Sv background. Mice carrying the deleted and floxed *Shox2* alleles were described previously [25], while mice carrying the *Nestin*-*Cre* transgene were from the Jackson Laboratory (Bar Harbor, ME), and described previously [35]. BAC RP23-105B3-*lacZ* transgenic animals were described previously [37], while *Shox2* ^*lacZ*^ mice are described below. All experiments performed at the University of Calgary were approved by the Life and Environmental Sciences Animal Care Committee. Experiments were conducted on three embryos or neonates of a particular genotype at each gestational and postnatal time point described, unless otherwise stated.

### Generation and characterization of *Shox2*^*lacZ*^ mice

*Shox2* ^*lacZ*^ mice were produced by pronuclear injection of a 15.4 kb *Nar*I fragment (Additional file 1: Figure S1A) of BAC RP23-103D17-*lacZ* that has a *lacZ* cassette inserted in the *Shox2* gene as previously described [37]. One of seven transgenic lines carrying this transgene showed an unexpected expression pattern that recapitulated all of the expression domains of the endogenous *Shox2* gene (compare Additional file 1: Figure S1D–S1E), despite evidence that the regulatory regions controlling expression in most of these domains is not within the transgene sequence [37]. This result suggested that the transgene in this line, designated *Shox2* ^*lacZ*^ may have inserted into the *Shox2* locus. Although rare, targeting of transgenic constructs generated by pronuclear injection has been reported [41, 42]. We confirmed a loss of *Shox2* function by a complementation test and targeting was shown by long range PCR (Additional file 1: Figure S1B). For the former, the *Shox2* ^*lacZ*^ allele was combined with the *Shox2* ^−^ allele. Mice with the genotype *Shox2* ^*lacZ/*−^ died at birth with defects similar to *Shox2* null animals, showing non-complementation (the limb and palate phenotypes are shown in Additional file 1: Figure S1F–K, respectively). Long-range PCR was performed with a forward primer corresponding to a sequence 4.5 kb upstream of *Shox2* (327 bp upstream of the 5′ limit of the transgene) and a reverse primer within the *lacZ* sequence. These primers amplified a 5.2 kb PCR product from DNA of S*hox2* ^*lacZ*^ mice as expected if the transgene were inserted at the *Shox2* genomic locus (Additional file 1: Figure S1B). Furthermore, western blotting failed to detect SHOX2 protein in lysates from E11.5 limb buds of *Shox2* ^*lacz/lacZ*^ embryos while lysates from wild-type littermate embryos showed clear SHOX2 signal (data not shown). Therefore we conclude that the *Shox2* ^*lacZ*^ allele is a null or severely hypomorphic allele. The precise molecular nature of the transgene insertion at its 3′ end has not yet been determined.

### X-gal staining

Embryos with *lacZ* transgenes were stained with X-gal according to standard techniques [43]. ImageJ was used to determine the relative size of the facial motor nucleus in *lacZ* stained control and *NestinCre; Shox2* ^*lacZ/flox*^ sagittal sections at E14.5 and P0 (Figure 4t–u).

### In situ hybridization

Whole-mount in situ hybridization (WISH) with a riboprobe was previously described [44]. In situ hybridization (ISH) on cryosections was previously described [25]. The *Shox2* [43], *Shh* [45] riboprobes have been described elsewhere. PCR primers used for generating the *Ptch1* riboprobe are as follows: Fwd: GGGAGGAAATGCTGAATAAAGCC and Rev: CCAGGAGGAAGACATCATCCACAC, while the primers used for generating the *Isl1, Cntn2, Tubb3, Periph, Slc18a3, Phox2b,* and *Slit2* riboprobes were obtained from the Allen brain atlas website (http://www.brain-map.org). DIG labeled riboprobes were detected with an AP-conjugated anti-DIG antibody (Roche) and BM Purple AP (Roche) was used as the color development substrate. All samples/slides used for comparisons were processed together. Following staining, tissue sections were mounted using Aqua Poly/Mount (Polysciences Inc.). ISH images were taken with a Leica MZ12.5 stereomicroscope using the associated Leica software. Brightness and/or contrast of the entire image was adjusted using Adobe Photoshop CS5.1 if deemed appropriate.

### Immunohistochemistry

Dissected embryos and postnatal brains were placed in ice-cold PBS, and fixed in 4% paraformaldehyde (PFA) overnight at 4°C. The tissue was washed in PBS and equilibrated in 30% sucrose/PBS overnight at 4°C. Both embryos and postnatal brains were embedded in Tissue Freezing Medium (TFM, Triangle Biomedical Sciences) and cryosectioned (16–20 μm sections). For immunohistochemistry (IHC), cryosections were rehydrated in PBS, washed with PBT (0.1% Triton-X), blocked using 5% normal goat serum (NGS) for 2 h at room temperature, and exposed to anti-active Caspase-3 (1:400, BD Pharmingen or 1:500, Promega) at 4°C overnight. Slides were then washed with PBT and exposed to secondary antibody (1:200, alexa 488 goat-anti-rabbit, Life Technologies) for 2 h at room temperature. Sections were mounted using Aqua Poly/Mount (Polysciences Inc.). Fluorescent IHC images were captured on a Zeiss LSM710 confocal microscope.

### Neurofilament staining

Antibodies recognizing neurofilaments (2H3) were used to visualize nerves in embryos or postnatal day 0 (P0) pup heads (skin was removed at P0). Samples were fixed at least 4 h in Dent’s fixative (4:1, Methanol:DMSO). Embryos and P0 heads were processed according to standard techniques previously described [26]. The 2H3 antibody was developed by T. Jessell/J. Dodd [46] and was obtained from the Developmental Studies Hybridoma Bank developed under the auspices of the NICHD and maintained by The University of Iowa, Department of Biology, Iowa City, IA 52242.

### Quantification methods and statistical analysis

Quantitative results for cell counts are represented by mean scores ± S.E.M. and were analyzed by two-tailed unpaired t tests using Prism 3 for Macintosh (GraphPad Software, La Jolla, CA, USA).

### Additional files

Additional file 1: **Figure S1.** *Shox2* ^*lacZ*^ transgenic analysis. (A) Schematic of the 15.4 kb *Shox2*-*LacZ* transgene (above) and the endogenous *Shox2* genomic locus (below). The five exons are shown as vertical boxes; red indicates the coding portion of the exons and unfilled regions indicate the untranslated regions (UTRs). Green boxes/arrows indicate the sequences and sizes (in basepairs) amplified by PCR in panels B and C. The 5,200 bp PCR product amplified from genomic DNA of *Shox2* ^*lacZ*^ mice (panel B) provides evidence for targeting via homologous recombination at the 5′ end of the gene (dotted lines). The forward primer for this PCR is 327 bp upstream of the transgene sequence and the reverse primer is within the *lacZ* cassette (blue). The 3′ insertion site has not yet been characterized. (B) Long range PCR demonstrating location of the transgene at the *Shox2* locus, as shown by the 5.2 kb PCR product in five mice carrying the transgene but not in wild-type controls. (C) Genotyping of *Shox2* ^*lacZ*^ mice. The wild-type allele is indicated by the 705 bp band and the transgene insertion is detected by the 392 band, with both bands amplified in heterozygote animals. The two lanes at right have only the lower band, indicating that homozygous *Shox2* ^*lacz/lacZ*^ mice do not have an intact exon 1 in their genomes, which further supports targeting at the *Shox2* locus. (D) WISH for *Shox2* at E12.5. (D’-D’’’) ISH for *Shox2* on E12.5 brain sections. (E) X-gal staining of an E12.5 *Shox2* ^*lacZ/*+^ embryo and corresponding brain sections (E’-E’’’). D’ and E’ are located anterior to D’’’ and E’’’. (F-I) Forelimb (F, G) and hindlimb (H, I) skeletons of newborn *Shox2* ^*lacZ/*−^ mice (G, I) and controls (F, H) showing reduction in the humerus (h) and femur (f) as those found in *Shox2*-null mice. (J, K) Hematoxylin and eosin (H&E) stained coronal sections through the palate of newborn *Shox2* ^*lacZ/*−^ mice (K) and controls (J) displays a cleft palate (compare J to K, dashed-box and arrow) similar to what is found in *Shox2*-null mice. Abbreviations: ey, eye; tg, trigeminal; di, diencephalon; mes, mesencephalon; hb, hindbrain; drg, dorsal root ganglia; nt, neural tube; fl, forelimb; hl, hindlimb; sc, scapula; r, radius; u, ulna; a, autopod; pg, pelvic girdle; t, tibia; fi, fibula; PS, palate shelf

Additional file 2: **Figure S2.** Conditional removal of *Shox2* using *Nestin*-*Cre* does not result in disruptions to the migratory programs of vMNs. (A) Diagram of a transverse section through rhombomere 4 (r4) of the E11 brain highlights progenitors (red arrow, known to be *Phox2b* + [18]) and post-mitotic neurons (black arrow, region known to be *Phox2b* +*/Isl1* + [18]) adjacent to the floor plate (fp) (image adapted from Pattyn et al. [18]). (B) ISH for *Shox2* on an E11 transverse brain section through rhombomere 4 shows *Shox2* expression in post-mitotic neurons (black arrow) but not in progenitors (red arrow). (C-E) ISH images from the Allen Brain Atlas database (http://www.brain-map.org) show *Shox2* (C, http://developingmouse.brainmap.org/experiment/siv?i100082467&imageId = 101400047&initImage = ish), *Isl1* (D, http://developingmouse.brain-map.org/experiment/siv?id = 100029096&imageId = 100647957&initImage = ish) and *Phox2b* (E, http://developingmouse.brain-map.org/experiment/siv?id=100077806&imageId=101287085&initImage=ish) expression in vMNs in sagittal sections at E11.5 (red arrows point to *Phox2b* + vMNs adjacent to the ventricular zone). (F-K) ISH on E11.5 serial sagittal sections through the hindbrain of control (F, H, J) and *Nestin*-*Cre; Shox2* ^*flox/*−^ mutant (G, I, K) embryos shows loss of *Shox2* expression in the brain (compare F to G, arrows), while *Isl1* (compare H to I, arrows) and *Phox2b* (compare J to K, arrows) expression is maintained in neurons migrating from the approximate rhombomere 4/5 (r4/r5) boundary to rhombomere 6 (r6), depicted using a red-dashed line. (L) Image of an E11.5 hindbrain sagittal section available from the Allen Brain Atlas database (http://atlas.brain-map.org/atlas?atlas=181275741#atlas= 181275741&plate = 100425904&structure = 126651910&x = 5432&y = 2371&zoom = -2&resolution = 3.96&z = 6) indicates rhombomere divisions (r2 to r7) within the pontomedullary hindbrain (PMH). (M-R) ISH on E12.5 serial sagittal sections through the hindbrain of control (M, O, Q) and *Nestin*-*Cre; Shox2* ^*flox/*−^ mutant (N, P, R) embryos shows loss of *Shox2* expression in the brain (compare M to N, arrow), while *Isl1* (compare O to P, arrow) and *Phox2b* (compare Q to R, arrow) expression remain (approximate r5/6 boundary depicted using a red-dashed line). (S) Image of an E13.5 hindbrain sagittal section available from the Allen Brain Atlas database (http://atlas.brainmap.org/atlas?atlas=181276130#atlas = 181276130&plate = 100793031&structure = 111220768&x = 4931&y = 2197&zoom = -2&resolution = 3.96&z = 6) indicates rhombomere divisions (r5 to r7). Scale bar = 500 μm

Additional file 3: **Figure S3.** Conditional removal of *Shox2* using *Nestin*-*Cre* does not result in disruptions in palate development. (A, B) WISH on E12.5 control (A) and *Nestin*-*Cre; Shox2* ^*flox/*−^ mutant (B) embryos shows loss of *Shox2* expression in parts of the developing trigeminal (V) ganglion (arrow), in addition to the developing maxillary process (mp, arrow) and mandibular arch (ma, arrow) in *Nestin*-*Cre; Shox2* ^*flox/*−^ mutants as compared to controls. (C, D) ISH on E12.5 sagittal sections through the hindbrain of control (C) and *Nestin*-*Cre; Shox2* ^*flox/*−^ mutant (D) embryos shows loss of *Shox2* expression in the brain, including the facial motor nucleus (nVII, dashed-circle). (E) Representative image of P0 control (left) and *Nestin*-*Cre; Shox2* ^*flox/*−^ mutant (right) pups shows *Nestin*-*Cre; Shox2* ^*flox/*−^ animals with less milk in their stomachs as compared to controls (red arrows). (F-I) Coronal sections through the palate of control (F, H) and *Nestin*-*Cre; Shox2* ^*flox/*−^ (G, I) pups demonstrate that *Nestin*-*Cre; Shox2* ^*flox/*−^ animals have an intact palate (compare F to G and H to I, arrows). F and G are rostral sections, while H and I are more caudal sections through the palate. (J, K) Representative P0 control (J) and *Nestin*-*Cre; Shox2* ^*flox/*−^ (K) pup palates (viewed ventrally) show that *Nestin*-*Cre; Shox2* ^*flox/*−^ mutant animals have an intact palate. Abbreviations: ey, eye; cb, cerebellum; PS, palate shelf; PP. Scale bar = 500 μm

Additional file 4: **Movie 1.** Representative video of a P0 control and *Nestin*-*Cre; Shox2* ^*flox/*−^ mutant pup. This video highlights the different levels of physical activity of *NestinCre*-*Cre; Shox2* ^*flox/*−^ mutant pups (which are less active and display tremors) as compared to control pups, in addition to demonstrating that *NestinCre*-*Cre; Shox2* ^*flox/*−^ mutant pups have very little milk in their stomachs as compared to controls

Additional file 5: **Figure S4.** Conditional removal of *Shox2* using *Wnt1*-*Cre* does not influence axonal projection properties of vMNs. (A, B) WISH on E12.5 control (A) and *Wnt1*-*Cre; Shox2* ^*flox/*−^ mutant (B) embryos show loss of *Shox2* expression in the developing trigeminal (V) ganglion, in addition to the developing maxillary process (mp) and mandibular arch (ma). (C, D) Side view of the E12.5 face of control (A) and *Wnt1*-*Cre; Shox2* ^*flox*/−^ mutant (B) embryos stained with the 2H3 anti-neurofilament antibody show intact facial nerves (VII). Abbreviations: ey, eye. Scale bar = 500 μm
