# Supplementary figures and images for: Shox2 is required for the proper development of the facial motor nucleus and the establishment of the facial nerves
Source: BMC Neurosci. 2015 Jul 9;16:39. doi: 10.1186/s12868-015-0176-0 (PMC4495855; doi:10.1186/s12868-015-0176-0)

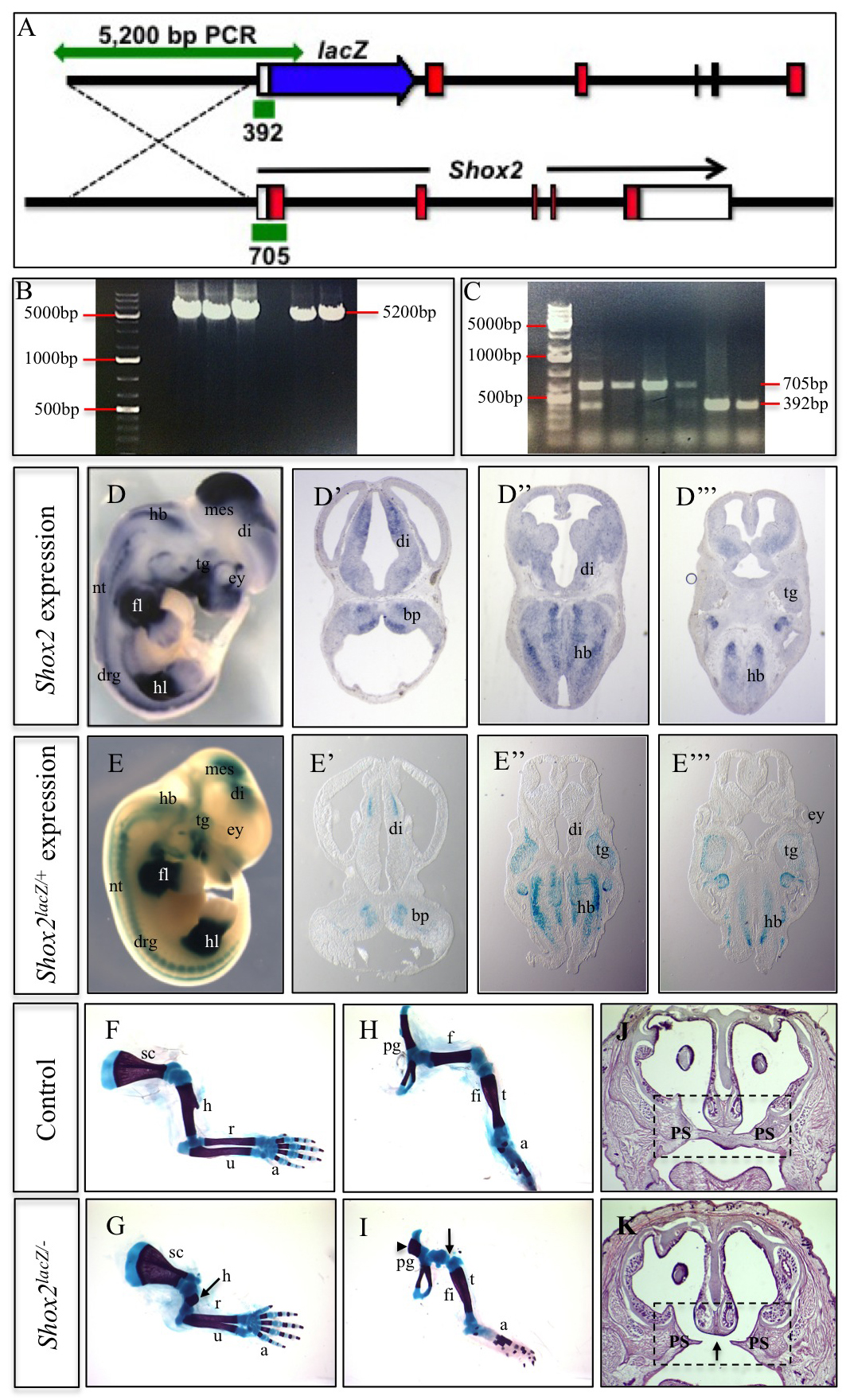

Supplement: Supplementary file 1 — Figure S1. Shox2 lacZ transgenic analysis. (A) Schematic of the 15.4 kb Shox2-LacZ transgene (above) and the endogenous Shox2 genomic locus (below). The five exons are shown as vertical boxes; red indicates the coding portion of the exons and unfilled regions indicate the untranslated regions (UTRs). Green boxes/arrows indicate the sequences and sizes (in basepairs) amplified by PCR in panels B and C. The 5,200 bp PCR product amplified from genomic DNA of Shox2 lacZ mice (panel B) provides evidence for targeting via homologous recombination at the 5′ end of the gene (dotted lines). The forward primer for this PCR is 327 bp upstream of the transgene sequence and the reverse primer is within the lacZ cassette (blue). The 3′ insertion site has not yet been characterized. (B) Long range PCR demonstrating location of the transgene at the Shox2 locus, as shown by the 5.2 kb PCR product in five mice carrying the transgene but not in wild-type controls. (C) Genotyping of Shox2 lacZ mice. The wild-type allele is indicated by the 705 bp band and the transgene insertion is detected by the 392 band, with both bands amplified in heterozygote animals. The two lanes at right have only the lower band, indicating that homozygous Shox2 lacz/lacZ mice do not have an intact exon 1 in their genomes, which further supports targeting at the Shox2 locus. (D) WISH for Shox2 at E12.5. (D’-D’’’) ISH for Shox2 on E12.5 brain sections. (E) X-gal staining of an E12.5 Shox2 lacZ/+ embryo and corresponding brain sections (E’-E’’’). D’ and E’ are located anterior to D’’’ and E’’’. (F-I) Forelimb (F, G) and hindlimb (H, I) skeletons of newborn Shox2 lacZ/− mice (G, I) and controls (F, H) showing reduction in the humerus (h) and femur (f) as those found in Shox2-null mice. (J, K) Hematoxylin and eosin (H&E) stained coronal sections through the palate of newborn Shox2 lacZ/− mice (K) and controls (J) displays a cleft palate (compare J to K, dashed-box and arrow) similar to what is found in Shox2-null [file 12868_2015_176_MOESM1_ESM.jpg]

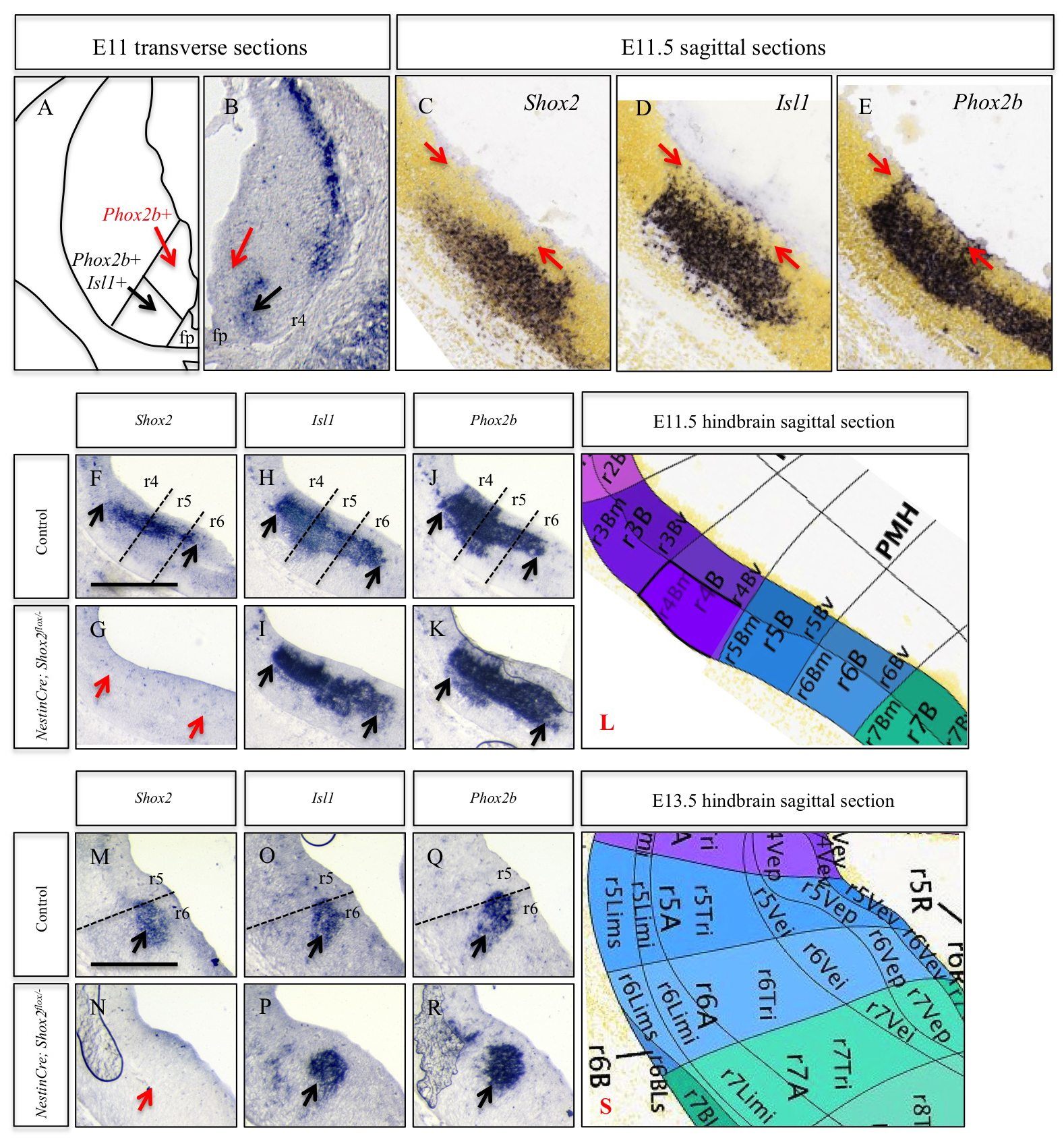

Supplement: Supplementary file 2 — Figure S2. Conditional removal of Shox2 using Nestin-Cre does not result in disruptions to the migratory programs of vMNs. (A) Diagram of a transverse section through rhombomere 4 (r4) of the E11 brain highlights progenitors (red arrow, known to be Phox2b + [18]) and post-mitotic neurons (black arrow, region known to be Phox2b +/Isl1 + [18]) adjacent to the floor plate (fp) (image adapted from Pattyn et al. [18]). (B) ISH for Shox2 on an E11 transverse brain section through rhombomere 4 shows Shox2 expression in post-mitotic neurons (black arrow) but not in progenitors (red arrow). (C-E) ISH images from the Allen Brain Atlas database (http://www.brain-map.org) show Shox2 (C, http://developingmouse.brainmap.org/experiment/siv?i100082467&imageId = 101400047&initImage = ish), Isl1 (D, http://developingmouse.brain-map.org/experiment/siv?id = 100029096&imageId = 100647957&initImage = ish) and Phox2b (E, http://developingmouse.brain-map.org/experiment/siv?id=100077806&imageId=101287085&initImage=ish) expression in vMNs in sagittal sections at E11.5 (red arrows point to Phox2b + vMNs adjacent to the ventricular zone). (F-K) ISH on E11.5 serial sagittal sections through the hindbrain of control (F, H, J) and Nestin-Cre; Shox2 flox/− mutant (G, I, K) embryos shows loss of Shox2 expression in the brain (compare F to G, arrows), while Isl1 (compare H to I, arrows) and Phox2b (compare J to K, arrows) expression is maintained in neurons migrating from the approximate rhombomere 4/5 (r4/r5) boundary to rhombomere 6 (r6), depicted using a red-dashed line. (L) Image of an E11.5 hindbrain sagittal section available from the Allen Brain Atlas database (http://atlas.brain-map.org/atlas?atlas=181275741#atlas= 181275741&plate = 100425904&structure = 126651910&x = 5432&y = 2371&zoom = -2&resolution = 3.96&z = 6) indicates rhombomere divisions (r2 to r7) within the pontomedullary hindbrain (PMH). (M-R) ISH on E12.5 serial sagittal sections through the hindbrain of control (M, O, Q) and Ne [file 12868_2015_176_MOESM2_ESM.jpg]

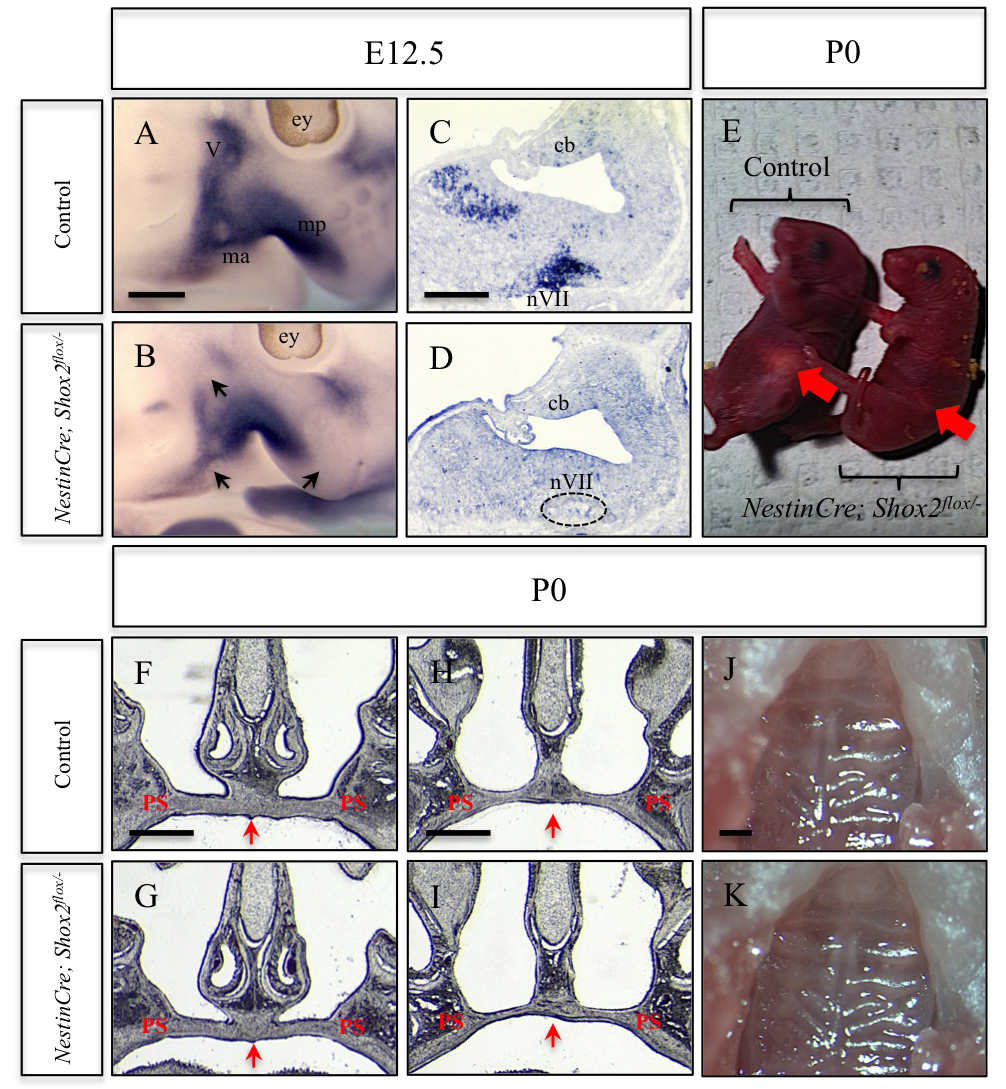

Supplement: Supplementary file 3 — Figure S3. Conditional removal of Shox2 using Nestin-Cre does not result in disruptions in palate development. (A, B) WISH on E12.5 control (A) and Nestin-Cre; Shox2 flox/− mutant (B) embryos shows loss of Shox2 expression in parts of the developing trigeminal (V) ganglion (arrow), in addition to the developing maxillary process (mp, arrow) and mandibular arch (ma, arrow) in Nestin-Cre; Shox2 flox/− mutants as compared to controls. (C, D) ISH on E12.5 sagittal sections through the hindbrain of control (C) and Nestin-Cre; Shox2 flox/− mutant (D) embryos shows loss of Shox2 expression in the brain, including the facial motor nucleus (nVII, dashed-circle). (E) Representative image of P0 control (left) and Nestin-Cre; Shox2 flox/− mutant (right) pups shows Nestin-Cre; Shox2 flox/− animals with less milk in their stomachs as compared to controls (red arrows). (F-I) Coronal sections through the palate of control (F, H) and Nestin-Cre; Shox2 flox/− (G, I) pups demonstrate that Nestin-Cre; Shox2 flox/− animals have an intact palate (compare F to G and H to I, arrows). F and G are rostral sections, while H and I are more caudal sections through the palate. (J, K) Representative P0 control (J) and Nestin-Cre; Shox2 flox/− (K) pup palates (viewed ventrally) show that Nestin-Cre; Shox2 flox/− mutant animals have an intact palate. Abbreviations: ey, eye; cb, cerebellum; PS, palate shelf; PP. Scale bar = 500 μm [file 12868_2015_176_MOESM3_ESM.jpg]

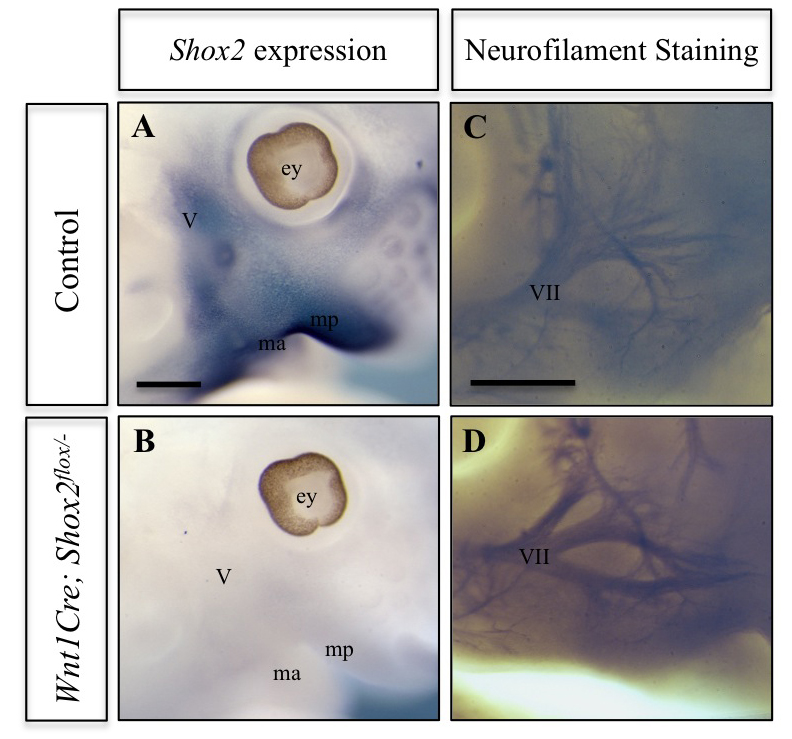

Supplement: Supplementary file 5 — Figure S4. Conditional removal of Shox2 using Wnt1-Cre does not influence axonal projection properties of vMNs. (A, B) WISH on E12.5 control (A) and Wnt1-Cre; Shox2 flox/− mutant (B) embryos show loss of Shox2 expression in the developing trigeminal (V) ganglion, in addition to the developing maxillary process (mp) and mandibular arch (ma). (C, D) Side view of the E12.5 face of control (A) and Wnt1-Cre; Shox2 flox/− mutant (B) embryos stained with the 2H3 anti-neurofilament antibody show intact facial nerves (VII). Abbreviations: ey, eye. Scale bar = 500 μm [file 12868_2015_176_MOESM5_ESM.jpg]
